# Supplementary material for: Evaluating a co-designed care bundle to improve patient safety at discharge from adult and adolescent mental health services (SAFER-MH and SAFER-YMH): protocol for a non-randomised feasibility study
Source: BMJ Open. 2023 Apr 11;13(4):e069216. doi: 10.1136/bmjopen-2022-069216 (PMC10106061; doi:10.1136/bmjopen-2022-069216)
Supplement: Author's manuscript [file bmjopen-2022-069216.draft_revisions.pdf]

BMJ Open

BMJ Open is committed to open peer review. As part of this commitment we make the peer review history of every article we publish publicly available.

When an article is published we post the peer reviewers' comments and the authors' responses online. We also post the versions of the paper that were used during peer review. These are the versions that the peer review comments apply to.

The versions of the paper that follow are the versions that were submitted during the peer review process. They are not the versions of record or the final published versions. They should not be cited or distributed as the published version of this manuscript.

BMJ Open is an open access journal and the full, final, typeset and author-corrected version of record of the manuscript is available on our site with no access controls, subscription charges or pay-per-view fees (http://bmjopen.bmj.com).

If you have any questions on BMJ Open's open peer review process please email info.bmjopen@bmj.com

Protocol for a non-randomised feasibility study evaluating a co-designed care bundle to improve patient safety at discharge from adult and adolescent mental health services (SAFER-MH and SAFER-YMH)

Journal:	*BMJ Open*
Manuscript ID	bmjopen-2022-069216
Article Type:	Protocol
Date Submitted by the Author:	23-Oct-2022
Complete List of Authors:	Tyler, Natasha; NIHR Greater Manchester Patient Safety Translational Research Centre; The University of Manchester, NIHR School for Primary Care Research<br>Angelakis, Ioannis; University of Liverpool, Department of Primary Care and Mental Health<br>Keers, Richard; The University of Manchester, Centre for Pharmacoepidemiology and Drug Safety Research<br>Planner, Claire; The University of Manchester, NIHR Greater Manchester Patient Safety Translational Research<br>hodkinson, alexander; University of Manchester, Centre for Primary Care<br>Giles, Sally; University of Manchester, NIHR Greater Manchester Primary Care Patient Safety Translational Research Centre<br>Grundy, Andrew; The University of Manchester<br>Kapur, Navneet; The University of Manchester, Centre for suicide prevention<br>Armitage, Chris; The University of Manchester, Manchester Centre for Health Psychology; The University of Manchester, NIHR Greater Manchester Patient Safety Translational Research Centre<br>Blakeman, Tom; The University of Manchester, NIHR Patient Safety Translational Research Centre; The University of Manchester, NIHR School for Primary Care Research<br>Campbell, Stephen; The University of Manchester, NIHR Greater Manchester Patient Safety Translational Research Centre<br>Robinson, Catherine; The University of Manchester<br>Leather, Jessica; The University of Manchester, NIHR Greater Manchester Patient Safety Translational Research Centre<br>Panagioti, Maria; The University of Manchester, NIHR Greater Manchester Patient Safety Translational Research Centre; The University of Manchester, NIHR School for Primary Care Research
Keywords:	MENTAL HEALTH, Child & adolescent psychiatry < PSYCHIATRY, Adult psychiatry < PSYCHIATRY

Protocol for a non-randomised feasibility study evaluating a co-designed care bundle to improve patient safety at discharge from adult and adolescent mental health services (SAFER-MH and SAFER-YMH)

Tyler, N.,[1,2,6]* Angelakis, I.,[3] Keers, R.N.,[4,5] Planner, C.,[1,2] Hodkinson, A.,[6] Giles, S.,[1,2] Grundy, A.[7], Kapur, N.[1,8,9], Armitage, C.J.A[1,10,11], Blakeman, T.,[1,2], Campbell, S.[2], Robinson, C.[12], Leather, J.[1,10], Panagioti, M.[1,2,6]

1. National Institute for Health and Care Research Greater Manchester Patient Safety Translational Research Centre, Division of Population Health, Health Services Research and Primary Care, University of Manchester, UK
2. Centre for Primary Care Research, School for Primary Care Research,_Division of Population Health, Health Services Research & Primary Care, University of Manchester, Manchester, UK
3. Institute of Population Health, Department of Primary Care and Mental Health, University of Liverpool, UK
4. Centre for Pharmacoepidemiology and Drug Safety, Division of Pharmacy and Optometry, University of Manchester, GM PSTRC
5. Suicide, Risk and Safety Research Unit, Greater Manchester Mental Health NHS Foundation Trust, Manchester
6. National Institute for Health and Care Research (NIHR) School for Primary Care Research, Division of Population Health, Health Services Research and Primary Care, School of Health Sciences, Faculty of Biology, Medicine and Health, Manchester Academic HealthScience Centre, University of Manchester, Manchester, UK
7. Division of Nursing, Midwifery & Social Work, School of Health Sciences, Faculty of Biology, Medicine, and Health, The University of Manchester
8. Centre for Mental Health & Safety, Division of Psychology & Mental Health, School of Health Sciences, Faculty of Biology, Medicine, and Health, The University of Manchester and Manchester Academic Health Sciences Centre, Manchester, UK
9. Greater Manchester Mental Health NHS Foundation Trust, Manchester, UK
10. Manchester Centre for Health Psychology, School of Health Sciences, University of Manchester, Manchester, UK
11. Manchester Academic Health Science Centre, Manchester University NHS Foundation Trust, Manchester, UK
12. Faculty of Biology, Medicine and Health, School of Health Sciences, University of Manchester, Manchester, UK

***Corresponding Author**
Dr. Natasha Tyler
Email address: natasha.tyler@manchester.ac.uk

For peer review only - http://bmjopen.bmj.com/site/about/guidelines.xhtml

Abstract

Introduction

Patients being discharged from inpatient mental wards often describe safety risks in terms of inadequate information sharing and involvement in discharge decisions. Through stakeholder engagement we co-designed, developed and adapted two versions of a care bundle intervention, the SAFER Mental Health care bundle for adult and youth inpatient mental health settings (SAFER-MH and SAFER-YMH respectively) that look to address these concerns through the introduction of new or improved processes of care.

Methods and analysis

Two uncontrolled before-and-after feasibility studies, where all participants will receive the intervention. We will examine the feasibility and acceptability of the SAFER-MH in inpatient mental health settings in patients aged 18 years or older who are being discharged and the feasibility and acceptability of the SAFER-YMH intervention in inpatient mental health settings in patients aged between 14 to 18 years who are being discharged. The baseline period and intervention periods are both 6 weeks. SAFER-MH will be implemented in three wards and SAFER-YMH in one or two wards, ideally across different trusts within England. We will use quantitative (e.g. questionnaires, completion forms) and qualitative (e.g. interviews, process evaluation) methods to assess the acceptability and feasibility of the two versions of the intervention. The findings will inform whether a main effectiveness trial is feasible and, if so, how it should be designed, and how many patients/wards should be included.

Ethics and dissemination

Ethical approval was obtained from the National Health Service Cornwall and Plymouth Research Ethics Committee and Surrey Research Ethics Committee (reference: 22/SW/0096 and 22/LO/0404). Research findings will be disseminated with participating sites and shared in a various ways to engage different audiences. We will present findings at international and national conferences, publish in open-access, peer-reviewed journals.

Strengths and limitations of this study (

- This is the first study evaluating a co-produced patient flow bundle for making discharge from acute inpatient mental health settings to the community safer and more patient-centred.
- A novel aspect of this study is that behaviour science techniques have been embedded in the components of the intervention for improving feasibility, acceptability, and user engagement.
- An in-depth mixed method approach (qualitative and quantitative) will be used to evaluate the study and inform the feasibility and key outcomes of the main trial.
- A potential limitation of this study is the lack of a control condition, although an extensive baseline phase is applied for comparing data before and after introducing the intervention.

For peer review only - http://bmjopen.bmj.com/site/about/guidelines.xhtml

Keywords

Feasibility Study
Protocol
Mental Health
Discharge
Care Transitions

Word count 3486

For peer review only

Introduction

It is estimated that one in four adults and one in ten children will be affected by mental or neurological disorders at some point in their lives (1,2). Mental illness has become the single largest cause of disability in England, costing the NHS over £13.3 billion in 2019/20 alone and is rising among both children and adults (3–5). Individuals that are admitted to inpatient mental health services are at higher risk of adverse outcomes such as shorter life expectancy; increased risk for homelessness; self-harm, violence and criminal activity; and increased likelihood of many other adverse psychosocial outcomes, including loneliness, isolation and stigma (6–10).

Transitions into and out of acute mental health hospitals are risky stages in the health care pathway associated with patient safety issues. The absence of continuity of care is an important risk factor for patient safety incidents during transitions of care (8). Other patient safety risk factors may include; difficulties of medication management across the care system; delayed discharge often arising from insufficient housing, social or community care provision and, lack of information sharing between services (primarily between primary and secondary care)(6,11–14).

Multi-faceted interventions have been increasingly used to improve patient safety and reduce readmissions in complex care transitions (11,15–17) . The SAFER Patient Flow Bundle is an exemplar multifaceted intervention, developed by NHS England/Improvement to improve

discharge from acute hospitals to the community. (18). The SAFER Patient Flow bundle consists of five components: 1) Senior review (before midday); 2) Expected discharge date and clinical criteria for discharge; 3) Early assessments to improve patient flow; 4) Early discharge (aiming to discharge patients before midday); and 5) Review a multi-disciplinary review for patients with increased lengths of stay (18). Many of the individual components of the SAFER bundle are used as standard practice or best practice guidelines in policy (19). Preliminary evidence obtained by case studies across the country is promising as it shows that SAFER has resulted in reduced length of patient stay in different hospitals, diminished discharge delays with minimal complications and decreased re-admissions or contact with primary care. Furthermore, patient and staff satisfaction have increased (20).

Although protocols, webinars, and/or other tools have been developed to facilitate the implementation of SAFER (20), there is currently lack of evidence examining its appropriateness within a mental health setting. To address this gap, we conducted a series of co-design empirical studies with multiple stakeholders including patients, carers, professionals and academics (21,22). These led to the development of two adapted versions of the SAFER patient flow bundle tailored to mental health, one for an adult acute mental health hospital (SAFER-MH) and one for Youth (Child and Adolescent) Mental Health Services (SAFER-YMH). Now that the bundle has been developed, we aim to generate data regarding its feasibility and acceptability, which will enrich the evidence base about its future wider adoption in NHS services.

The primary objectives of this research are therefore to:

1. examine whether the SAFER-MH and SAFER-YMH bundles are feasible and acceptable (based on standardised quantitative measures, and qualitative data) to patients, carers and professionals,
2. identify specific principle(s) of SAFER-MH and SAFER-YMH which need to be adapted to make it more acceptable, and increase its prospect of long-term implementation
3. examine the adherence to the delivery of the intervention (measured using a purposefully designed self-report form and recruitment rates), completion rate and appropriateness of the outcome measures and recruitment strategies
4. explore whether healthcare professional and patient engagement with the SAFER-MH and SAFER-YMH interventions can be increased using evidence-based behavioural science techniques

Methods and Analysis

Study design

This study will include two concurrent distinct studies, receiving adapted versions of the same intervention:

- Phase 1: SAFER-Mental Health (MH) for adults and,
- Phase 2: SAFER-Youth Mental Health (YMH) for children and adolescents which includes an adaption of the original SAFER-MH for young people.

These will be uncontrolled before-and-after feasibility studies where all participants will receive the intervention. We will use quantitative (e.g. questionnaires, completion forms) and qualitative (e.g. interviews, process evaluation) methods to assess acceptability and feasibility.

Intervention

The SAFER-MH is a co-designed adapted version of the SAFER Patient Flow Bundle (NHS Improvement) intervention (20) and will be delivered to patients as part of their normal care pathway. The intervention was adapted based on 35 stakeholder interviews and RAND consensus methods with multi-disciplinary experts and professionals (21,22). SAFER-YMH involved a further level of co-design to ensure that the intervention is suitable for adolescent

For peer review only - http://bmjopen.bmj.com/site/about/guidelines.xhtml

services. This involved presenting the adult version to professional and patients' groups to discuss relevant changes to be made to make it more appropriate for adolescent services. First, we aim to identify the elements of existing practice in the pilot sites before implementing the SAFER interventions, in both studies. The intervention focuses on promoting best practice guidance and has three key stages: Admission, Discharge and Weekly Tasks and are discussed below:

1. Admission
At admission, the intervention group will complete three key tasks: 1) Setting criteria for discharge and an estimated discharge day, 2) Identifying early social information that will help plan for discharge and, 3) Introducing the Patient Written Discharge Plan. This will be structured within two documents including the admissions social information capture document and the transitions checklist, which will be completed at multiple times in the patient journey.

2. Weekly Tasks
There are three weekly tasks that will form part of this assessment 1) Senior review of discharge readiness, 2) Multi-disciplinary discharge team meeting and, 3) Multi-agency discharge team meeting.

3. Discharge
At discharge, there will be two key tasks 1) Co Producing a high-quality patient written discharge plan and, 2) Ensuring the patient has their copy of the patient written discharge plan.

Participants

All patients admitted to pilot wards during the study period (14 weeks from November 2022 onwards) will receive the intervention, as it is a best practice intervention delivered as a service improvement change in care. Each individual participant will then be given the option to consent (parental/guardian consent for under 16s) whether to be a participant in the research studies by participating in the interviews and by completing relevant questionnaires. Patient participants will be adults (phase 1) or young people (phase 2) with mental illness who were admitted to an inpatient ward. Healthcare professionals that will be delivering the intervention and carers/family members of the patients (who consent to be involved) will also be involved in the interview components of the study (patient and carers interviewees can decide whether to be interviewed alone or together).

Eligibility criteria

Eligible sites
Eligible sites will acute adult inpatient mental health wards. As this is a feasibility study will aim to test on a variety of wards (i.e., female, male or mixed).

Phase 1: SAFER-MH
The eligibility criteria for individual patient participants to take part in the SAFER-MH study reflects the eligibility criteria for services. We will include adult patients aged 18-65 years, informal carers for adult patients with mental health problems aged over 18, healthcare and professionals that are involved in delivering the intervention and informal carers of patients (for process evaluation). All patients will receive the service-level intervention but will choose to participate in the study if they feel they have the capacity consent.

Phase 2: SAFER-YMH
The eligibility criteria to take part in the feasibility study reflects the eligibility criteria for services. Any young person accessing inpatient CAMHS services would be eligible to

For peer review only

participate, a parent/carer of a young person accessing inpatient CAMHS services and healthcare Professionals that are involved in delivering the intervention and parents/guardians of the children. All patients will receive the service-level intervention but will choose to participate in the study if they (their parents/guardians) feel they have the capacity and consent.

Recruitment and Data Collection

Inpatient mental health ward sites

Existing networks will be used to identify eligible services (wards) for the SAFER-MH/YMH studies. The study team will meet with identified services (face-to-face or remotely) to fully explain the purposes of the studies and describe their requirements. Informed consent for services to participate will be provided by the research lead/authorised person in each service, acting as 'guardian' for patients in their care. This will follow agreement with each local ward team clarifying willingness to undertake the SAFER-MH/YMH interventions. Service consent to participate in the study will be formalised through written agreements. The number of services approached, declining, or considered not eligible will be recorded.

Phase 1: SAFER-MH We aim to recruit three pilot inpatient wards across different trusts in the north west of England to reflect variation of NHS services in England.

Phase 2: SAFER-YMH

We aim to recruit one or two pilot inpatient wards within one trust in the North-West of England. We do not aim to recruit additional trusts because due to funding restrictions, phase 2 must be completed by March 2023.

Patient Participants

All patients on a participating ward will receive the intervention as it is a service level change. However, we will identify patients that are willing to participate in the study assessments (questionnaires and interviews). A ward co-ordinator (e.g., nurse or other health professional) working within the participating services will be asked to co-ordinate handing out questionnaire packs to eligible patients. Potentially eligible patients will be assigned an individual study ID and given an invitation pack close to their expected discharge. This includes an Invitation letter, Patient Information Sheet (PIS), and a Consent to Contact form.

On receipt of an invitation pack, the potential participant will complete the included documents and return to the research practitioner on site. Consent for researchers to contact the potential participant regarding the study will be obtained, in line with the definition outlined in Article 4(11) of the GDPR guidance, "any freely given, specific, informed and unambiguous indication of the data subject's wishes by which he or she, by a statement or by a clear affirmative action, signifies agreement to the processing of personal data relating to him or her" (European Union, 2016). Consent to be contacted with a study recruitment pack will therefore be implied by completion and return of a completed Consent to Contact form.

Assent for young patient participants

Assent will be sought from children / young persons' parents/guardians, or others who are not legally empowered to give consent in phase 2.

Carer/parent/guardian Participants

For peer review only - http://bmjopen.bmj.com/site/about/guidelines.xhtml

A study pack will be handed to each patient at discharge, this will include a carer pack, which they can choose to pass on or discard. The pack will include details of the study, paper questionnaires and a paper consent to contact form for interviews, also an online link to assess the documents remotely. For patients who agree to be contacted for an interview, we will ask again if they would like to share the information pack and contact details with their informal carer when contacted.

Professional Participants

Staff participants will be those working directly with patient participants on participating mental health wards (i.e. nurses, health care assistants and doctors). Staff will self-identify to be involved in interviews and questionnaires through posters on the ward and emails sent by the trust research clinician/gatekeeper containing the study information pack (PIS, invitation letter, consent to contact form). All staff that complete the training will be given the opportunity to complete the evaluation survey.

Procedure
Pre-intervention (baseline) phase

This phase will consist of the usual care that the patients in the inpatient wards receive and will last for 6 weeks (see Table 1). We will assess this current practice by conducting interviews and by inspecting current documentation sheets. These will be completed by one on-site research practitioner. We will perform individual online interviews with the patients, mental health professionals, and carers (relatives) to assess the degree of the satisfaction of the patients and carers as well as their views/thoughts regarding their current discharge plans. At the end of this phase, patients will be asked to complete a series of study questionnaires, including the EQUIP PROM scale (23), which measures the involvement of both the patients and carers in health care planning during/after discharge, The Care Transitions Measure (CTM) (24), and a five-item patient-centred scale focusing on mental health discharge outcomes that were identified as important to patients in previous work (9). Patients, carers, and professionals will also be invited to be interviewed about their experiences of usual care, via the study information pack.

Table 1: Data collection procedures

Patient/Carer Outcome Measures (n=45):<br>- EQUIP-PROM scale, and,<br>- 5-items regarding general satisfaction about discharge planning	Patient/Carer Outcome Measures (n=45):<br>• EQUIP-PROM scale,<br>• 5-items regarding general satisfaction, and<br>• the Care Transitions Measure (CTM)<br><br>Staff Outcome Measures (n=45):<br>• AIM<br>• IAM<br>• FIM<br>• Adherence to the delivery of the Safer patient flow bundle will be measured

For peer review only

For peer review only - http://bmjopen.bmj.com/site/about/guidelines.xhtml

- using self-report questionnaires

Training phase

The training will be a series of self-led short videos and self-help manuals to the pilot ward staff participating in the study. This phase will last for 2 weeks. We aim to use evaluation questionnaires following the training. Interviews in the training phase will explore whether the interventions have been fully comprehended by staff and ensure that we have enough time to solve any problems regarding the training as they arise.

Intervention phase

This phase will last for 6 weeks during which the wards will receive the SAFER-MH and SAFER-YMH patient flow bundle (figure 1). Data regarding the adherence of the mental health professionals to using the SAFER-MH bundle will be collected. Individual online interviews (Zoom) will be conducted with patients and carers (within three months after discharge), and with mental health professionals (within one month after the completion of the 6-week intervention phase) to assess the feasibility and acceptability of the SAFER–MH bundle. Each patient/carer will only be interviewed once, professionals may be interviewed more than once in different stages of the project. At the end of this 6-week phase, patients will be asked to complete the same study scales that were administered during the baseline and which were completed by a different pool of patient participants discharged at the time. We will also administer the three staff outcome measures after the intervention phase is complete.

Figure 1: Timeline of events

Outcomes

The aim of the evaluation will be to determine the feasibility and acceptability of the SAFER-MH and SAFER-YMH interventions by patients, carers and professionals. Additionally, we aim to identify the completion rates and if there are any changes in the key outcomes. As this is a pre-post uncontrolled feasibility study, it will only give us a first indication about the appropriateness of the outcome measures whereas we will primarily focus on whether the SAFER-MH and SAFER-YMH is acceptable and, if so, for whom.

Quantitative Outcomes

These will be measured in the baseline phase and intervention phase with different patients. To assess patients and carers perspectives, the quantitative outcomes will be measured using paper/online versions of a series of questionnaires which include: 1) the EQUIP patient reported outcome measure, a 14-item co-designed measure to assess patient/carer views of care planning in mental health (23); 2) a short 4-item version of the Care Transitions Measure (CTM-4) (24), and 3) the five novel items assessing the safety of mental health care transitions (9).

For peer review only - http://bmjopen.bmj.com/site/about/guidelines.xhtml

To assess professional perspectives, we will use three measures the Acceptability, Appropriateness and Feasibility of Intervention Measure (AIM, IAM and FIM). Each of the three measure contains 4-items to assess professionals' perspectives of a new intervention. These measures will be administered after the intervention phase only (not the baseline). Adherence to the delivery of the SAFER-MH and SAFER-YMH bundle will be measured using a purposefully designed self-report form and recruitment rates will be recorded at each stage.

In-depth Qualitative Outcome data

A qualitative process evaluation will be conducted using semi-structured interviews to provide understanding of the study delivery and its potential scale-up. All participants that complete questionnaires will be given the opportunity to volunteer to be interviewed. We aim to interview patients and carers at baseline and intervention, whereas professionals will be interviewed at all three stages (baseline, training, and intervention). All participants (including children) will be remunerated in vouchers for participation.

Data Analysis Plan

We will be following the published extension to Consolidated Standards of Reporting Trials (CONSORT) (27) when reporting the results of this feasibility study (26) . As this is a feasibility study, no formal sample size calculation has been conducted. Our targeted sample size (n=90, phase 1, n=40, phase 2) should be large enough to consider the practicalities of recruitment and delivering the interventions.
Descriptive statistics for intervention feasibility, acceptability and appropriateness will be presented. A chart of participant flow during the whole study will be plotted (CONSORT flow chart) which will include information on the retention/withdrawal (including timing and reasons) of wards during the evaluation. We will also present the number of health practitioners who completed the questionnaires and interviews against the number of professionals working in the wards during the evaluation, and the number of patients/carers completing the questionnaires/interviews against those discharged during the duration of the study. We will also conduct basic psychometric analyses to verify the scale structure and internal consistency of the used questionnaires. For explorative purposes, we will examine differences between the three measures completed by patients recruited during the baseline phase and the measures completed by the patients during the intervention phase. Before starting with the analyses, we will examine if the data is normally distributed. If the data are normally distributed, parametric tests will be used, otherwise non-parametric tests will be used. The significance level for all analyses will be $p \le 0.05$. The data analyst will be blinded to the phase allocation (baseline or intervention phase).

Qualitative Analysis

We will use a framework approach to qualitative analysis, to inductively code and organise the data and identify emerging themes from the interviews. The frameworks will be based on two underpinning theoretical frameworks commonly used in similar research: the Conceptual Framework for Implementation Fidelity (5 domains), Theoretical Framework of Acceptability (29) and the Theoretical Domains Framework (14 domains). The coding process will follow these steps:

1. Familiarisation (researchers emerging themselves in the data),

2. Identifying a thematic framework (to generate an index of data based on a priori questions and issues raised by participants),

For peer review only - http://bmjopen.bmj.com/site/about/guidelines.xhtml

3. Indexing (applying thematic framework to the data using NVivo software),

4. Charting (Rearranging data in line with thematic framework), and

5. Mapping and interpretation (Using the charts to define concepts, map the range and nature of phenomena and find associations between themes).

Integration of quantitative and qualitative data

The quantitative and qualitative results will be initially considered separately but we aim to examine how they converge or differ when presenting the overall conclusions (28). Furthermore, the qualitative data collected across the two studies will be used to help explain or elaborate on the quantitative data (28).

Patient and public involvement

This study has a patient and public involvement service-user co-researcher who has been involved in the design and delivery of the study (AG). Patient and public involvement has been integral to this study and the development of the intervention which has been based on a series of stakeholder consultations, workshops and consensus methods (9,10,21,23). A core group of five patient and public involvement representatives form a steering committee for this study.

Ethics & Dissemination

Ethical approval was obtained from the National Health Service Cornwall and Plymouth Research Ethics Committee and Surrey Research Ethics Committee (reference: 22/SW/0096 and 22/LO/0404, respectively).

The study findings will be shared with participating inpatient sites in a range of ways to engage different audiences, as well as via presentations at national and international conferences and publishing in open-access peer-reviewed journals. Findings will be presented at a workshop for patients and carers a vlog will be created.

Discussion

This study aims to understand how to improve information sharing and capture between services during patient discharge from mental health hospitals to community services. Extensive stakeholder engagement with patients, carers, mental health professionals and academics has informed the intervention refinement and study co-design. We propose a pragmatic approach to test the feasibility and acceptability of a novel intervention which will add value to the evidence base about how information sharing/capture and patient/carer knowledge and involvement around discharge improves patient safety, health care utilisation and other important patient outcomes.

The findings will have to be interpreted and evaluated considering the limitations of the study design. For example, this is a pre-post uncontrolled study and the potential influence of inpatient settings cannot be controlled for through randomisation. Another limitation is that the interventions have only been developed in English and future work will need to explore how to culturally adapt it. Furthermore, some elements of the intervention may not be suitable for individuals with dementia who often occupy mental health inpatient beds (29), however funding has been obtained to develop this in future work. Finally, the intervention sites will be identified through collaborators, so selection bias must be considered.

For peer review only - http://bmjopen.bmj.com/site/about/guidelines.xhtml

Improving safety at mental health discharge is a key concern for patients, professionals and policy makers. Therefore, interventions aiming to improve knowledge sharing, shared decision-making and involvement of patients/carers are considered preferable (10,23,30). This study will determine the feasibility and acceptability of a multicomponent intervention to improve involvement of patients and/or carers in discharge planning. This will provide evidence for the acceptability of the SAFER-MH and SAFER-YMH to inform future improvements to scale to a randomised controlled trial, with concurrent process evaluation and economic evaluation.

Authors' contributions: NT and MP had the initial research idea and obtained funding for this study. NT, MP, and IA formulated the research questions and designed the study. AH NT, IA and MP devised the qualitative and statistical analysis plan. NT, MP and IA drafted the protocol. All author substantially contributed to the manuscript by providing review comments and edits and approved the final manuscript.

Funding statement: This work is supported by the NIHR Greater Manchester Patient Safety Translational Research Centre (grant number: PSTRC-2016-003) and NIHR School for Primary Care Research Capacity award 21/22 (grant number C015)Postdoctoral fellowship award. The work. The views expressed are those of the author(s) and not necessarily those of the
NIHR or the Department of Health and Social Care.

Competing interests statement. None

Acknowledgements

We would like to thank all of the patient, public, carer and professional stakeholders who have been involved in the co-design and development of the SAFER-MH intervention to date.

References

1. Mcmanus S, Meltzer H, Brugha T, Bebbington P, Jenkins R. Adult psychiatric morbidity in England-2007: Results of a household survey. Available from: https://digital.nhs.uk/data-and-information/publications/statistical/adult-psychiatric-morbidity-survey/adult-psychiatric-morbidity-in-england-2007-results-of-a-household-survey [Accessed on 08 August 2022]
2. NHS England: Mental health. Available from: https://www.england.nhs.uk/mental-health/ [Accessed on 08 August 2022]
3. Ford T, John A, Gunnell D. Mental health of children and young people during pandemic. *BMJ* 2021; 372: n614. doi.org/10.1136/bmj.n614
4. Newlove-Delgado T, McManus S, Sadler K, Thandi S, Vizard T, Cartwright C, et al. Child mental health in England before and during the COVID-19 lockdown. *The Lancet Psychiatry* 2021; 1: 8(5):353–4. doi.org/10.1016/S2215-0366(20)30570-8
5. British Medical Association. The impact of the pandemic on population health and

For peer review only - http://bmjopen.bmj.com/site/about/guidelines.xhtml

health inequalities. BMA Covid Review 5. Available from: https://www.bma.org.uk/advice-and-support/covid-19/what-the-bma-is-doing/the-impact-of-the-pandemic-on-population-health-and-health-inequalities Accessed on 08 August 2022]

6. Forchuk C, Russell G, Kingston-Macclure S, Turner K, Dill S. From psychiatric ward to the streets and shelters. *J Psychiatr Ment Health Nurs* 2006;13(3):301–8.
7. Walter F, Carr MJ, Mok PLH, Antonsen S, Pedersen CB, Appleby L, et al. Multiple adverse outcomes following first discharge from inpatient psychiatric care: a national cohort study. *The Lancet Psychiatry* 2019 1;6(7):582–589. doi: 10.1111/j.1365-2850.2006.00954.x.
8. Healthcare Quality Improvement Partnership. National Confidential Inquiry into Suicide and Safety in Mental Health. Annual report: England, Northern Ireland, Scotland and Wales. Available from: https://www.research.manchester.ac.uk/portal/en/projects/national-confidential-inquiry-into-suicide-and-safety-in-mental-health(788f9475-cadb-4697-bb82-817638044b7b).html Accessed on 08 August 2022]
9. Tyler N, Wright N, Grundy A, Waring J. Developing a core outcome set for interventions to improve discharge from mental health inpatient services: A survey, Delphi and consensus meeting with key stakeholder groups. *BMJ Open* 2020; **10:** e034215. doi:10.1136/bmjopen-2019-034215
10. Tyler N, Wright N, Panagioti M, Grundy A, Waring J. What does safety in mental health care transitions mean for service users and other stakeholder groups: An open-ended questionnaire study. *Heal Expect.* 2020; 24 Suppl 1(Suppl 1):185-194. doi: 10.1111/hex.13190
11. Tyler N, Wright N, Waring J. Interventions to improve discharge from acute adult mental health inpatient care to the community: systematic review and narrative synthesis. *BMC Health Serv Res* 2019 25;19(1):883. doi.org/10.1186/s12913-019-4658-0
12. Steffen S, Kösters M, Becker T, Puschner B. Discharge planning in mental health care: A systematic review of the recent literature. *Acta Psychiatrica Scandinavica* 2009; 120(1):1-9. doi: 10.1111/j.1600-0447.2009.01373.x.
13. Bonsack C, Golay P, Gibellini Manetti S, Gebel S, Ferrari P, Besse C, Favrod J, Morandi S. Linking primary and secondary care after psychiatric hospitalization: Comparison between transitional case management setting and routine care for common mental disorders. *Front Psychiatry* 2016;7: 96. doi: 10.3389/fpsyt.2016.00096.
14. Keers RN, Williams SD, Vattakatuchery JJ, Brown P, Miller J, Prescott L, et al. Medication safety at the interface: evaluating risks associated with discharge prescriptions from mental health hospitals. *J Clin Pharm Ther* 2015; 40(6): 645–54. doi: 10.1111/jcpt.12328
15. Gonçalves-Bradley DC, Lannin NA, Clemson L, Cameron ID, Shepperd S. Discharge planning from hospital. *Cochrane Database Syst Rev* 2022; (2).
16. Coffey A, Leahy-Warren P, Savage E, Hegarty J, Cornally N, Day MR, et al. Interventions to Promote Early Discharge and Avoid Inappropriate Hospital (Re)Admission: A Systematic Review. *Int J Environ Res Public Health* 2019;16(14). doi: 10.3390/ijerph16142457.
17. Leppin AL, Gionfriddo MR, Kessler M, Brito JP, Mair FS, Gallacher K, et al. Preventing 30-Day Hospital Readmissions: A Systematic Review and Meta-analysis of Randomized Trials. *JAMA Intern Med* 2014;174(7):1095–107. doi: 10.1001/jamainternmed.2014.1608.
18. NHS Improvement. Rapid improvement guide: the SAFER patient flow bundle. 2016. Available from: https://improvement.nhs.uk/resources/rapid-improvement-guide-safer-patient-flow-bundle/[Accessed on 08 August 2022]
19. National Institute for Health and Care Excellence. Transition between inpatient mental health settings and community or care home settings. Guidance and guidelines.

For peer review only

Available from: https://www.nice.org.uk/guidance/ng53 [Accessed on 08 August 2022]

20. The SAFER Patient Flow Bundle. Fab NHS Stuff. Available from: https://fabnhsstuff.net/fab-stuff/28574-the-safer-patient-flow-bundle [Accessed on 08 August 2022]
21. Tyler N, Planner C, Byrne M, Blakeman T, Keers RN, Wright O, et al. Developing Best Practice Guidance for Discharge Planning Using the RAND/UCLA Appropriateness Method. *Front Psychiatry* 2021;12: 2204. doi.org/10.3389/fpsyt.2021.789418
22. Tyler N. Daker-White G, Grundy A, Quinliivan L, Armitage C J, Campbell S, Panagioti M. The effects of the Covid-19 lockdown on quality and safety in mental health care transitions in England (In Press). *BJPsych Open* 2021;
23. Bee P, Gibbons C, Callaghan P, Fraser C, Lovell K. Evaluating and Quantifying User and Carer Involvement in Mental Health Care Planning (EQUIP): Co-Development of a New Patient-Reported Outcome Measure. *PLoS One* 2016; 11(3): e0149973. doi: 10.1371/journal.pone.0149973
24. Flink M, Tessma M, Småstuen MC, Lindblad M, Coleman EA, Ekstedt M. Measuring care transitions in Sweden: validation of the care transitions measure. *Int J Qual Heal Care* 2018;30(4):291. doi: 10.1093/intqhc/mzy001
25. Saint-Raymond A, Hill S, Martines J, Bahl R, Fontaine O, Bero L. Consort 2010. *The Lancet* 2010.
26. Lancaster GA, Thabane L. Guidelines for reporting non-randomised pilot and feasibility studies. *Pilot Feasibility Stud* 2019; 5(1):1–6. doi.org/10.1186/s40814-019-0499-1
27. Sekhon M, Cartwright M, Francis JJ. Acceptability of healthcare interventions: an overview of reviews and development of a theoretical framework. *BMC Heal Serv Res* 2017; 17(1):1–13. doi.org/10.1186/s12913-017-2031-8
29. Creswell JW. A Concise Introduction to Mixed Methods Research SAGE Publications; 2014. 152 p. Available from: https://books.google.com/books?hl=en&lr=&id=51UXBAAAQBAJ&pgis=1[Accessed on 08 August 2022]
29. Edmans BG, Wolverson E, Dunning R, Slann M, Russell G, Crowther G, et al. Inpatient psychiatric care for patients with dementia at four sites in the United Kingdom. *Int J Geriatr Psychiatry* 2022 ;37(2). doi: 10.1002/gps.5658
30. Slade M. Implementing shared decision making in routine mental health care. *World Psychiatry* 2017;16(2):146. doi: 10.1002/wps.20412

For peer review only

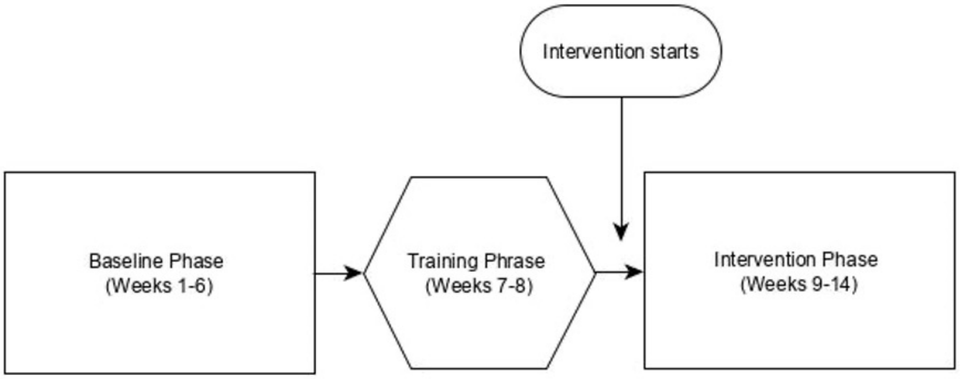

Figure 1: Timeline of events

159x66mm (144 x 144 DPI)

BMJ Open

Protocol for a non-randomised feasibility study evaluating a co-designed care bundle to improve patient safety at discharge from adult and adolescent mental health services (SAFER-MH and SAFER-YMH)

Journal:	*BMJ Open*
Manuscript ID	bmjopen-2022-069216.R1
Article Type:	Protocol
Date Submitted by the Author:	19-Jan-2023

Complete List of Authors:	Tyler, Natasha; NIHR Greater Manchester Patient Safety Translational Research Centre; The University of Manchester, NIHR School for Primary Care Research<br>Angelakis, Ioannis; University of Liverpool, Department of Primary Care and Mental Health<br>Keers, Richard; The University of Manchester, Centre for Pharmacoepidemiology and Drug Safety Research<br>Planner, Claire; The University of Manchester, NIHR Greater Manchester Patient Safety Translational Research<br>hodkinson, alexander; University of Manchester, Centre for Primary Care<br>Giles, Sally; University of Manchester, NIHR Greater Manchester Primary Care Patient Safety Translational Research Centre<br>Grundy, Andrew; The University of Manchester<br>Kapur, Navneet; The University of Manchester, Centre for suicide prevention<br>Armitage, Chris; The University of Manchester, Manchester Centre for Health Psychology; The University of Manchester, NIHR Greater Manchester Patient Safety Translational Research Centre<br>Blakeman, Tom; The University of Manchester, NIHR Patient Safety Translational Research Centre; The University of Manchester, NIHR School for Primary Care Research<br>Campbell, Stephen; The University of Manchester, NIHR Greater Manchester Patient Safety Translational Research Centre<br>Robinson, Catherine; The University of Manchester<br>Leather, Jessica; The University of Manchester, NIHR Greater Manchester Patient Safety Translational Research Centre<br>Panagioti, Maria; The University of Manchester, NIHR Greater Manchester Patient Safety Translational Research Centre; The University of Manchester, NIHR School for Primary Care Research

**Primary Subject Heading**:	Mental health
Secondary Subject Heading:	Evidence based practice
Keywords:	MENTAL HEALTH, Child & adolescent psychiatry < PSYCHIATRY, Adult

	psychiatry < PSYCHIATRY

For peer review only - http://bmjopen.bmj.com/site/about/guidelines.xhtml

Protocol for a non-randomised feasibility study evaluating a co-designed care bundle to improve patient safety at discharge from adult and adolescent mental health services (SAFER-MH and SAFER-YMH)

Tyler, N.,[1,2,6]* Angelakis, I.,[3] Keers, R.N.,[4,5] Planner, C.,[1,2] Hodkinson, A.,[6] Giles, S.,[1,2] Grundy, A. [7], Kapur, N. [1,8,9], Armitage, C.J.A [1,10,11], Blakeman, T.,[1,2], Campbell, S. [2], Robinson, C. [12], Leather, J. [1,10], Panagioti, M.[1,2,6]

1. National Institute for Health and Care Research Greater Manchester Patient Safety Translational Research Centre, Division of Population Health, Health Services Research and Primary Care, University of Manchester, UK
2. Centre for Primary Care Research, School for Primary Care Research,_Division of Population Health, Health Services Research & Primary Care, University of Manchester, Manchester, UK
3. Institute of Population Health, Department of Primary Care and Mental Health, University of Liverpool, UK
4. Centre for Pharmacoepidemiology and Drug Safety, Division of Pharmacy and Optometry, University of Manchester, GM PSTRC
5. Suicide, Risk and Safety Research Unit, Greater Manchester Mental Health NHS Foundation Trust, Manchester
6. National Institute for Health and Care Research (NIHR) School for Primary Care Research, Division of Population Health, Health Services Research and Primary Care, School of Health Sciences, Faculty of Biology, Medicine and Health, Manchester Academic HealthScience Centre, University of Manchester, Manchester, UK
7. Division of Nursing, Midwifery & Social Work, School of Health Sciences, Faculty of Biology, Medicine, and Health, The University of Manchester
8. Centre for Mental Health & Safety, Division of Psychology & Mental Health, School of Health Sciences, Faculty of Biology, Medicine, and Health, The University of Manchester and Manchester Academic Health Sciences Centre, Manchester, UK
9. Greater Manchester Mental Health NHS Foundation Trust, Manchester, UK
10. Manchester Centre for Health Psychology, School of Health Sciences, University of Manchester, Manchester, UK
11. Manchester Academic Health Science Centre, Manchester University NHS Foundation Trust, Manchester, UK
12. Faculty of Biology, Medicine and Health, School of Health Sciences, University of Manchester, Manchester, UK

***Corresponding Author**
Dr. Natasha Tyler
Email address: natasha.tyler@manchester.ac.uk

For peer review only - http://bmjopen.bmj.com/site/about/guidelines.xhtml

Abstract

Introduction

Patients being discharged from inpatient mental wards often describe safety risks in terms of inadequate information sharing and involvement in discharge decisions. Through stakeholder engagement we co-designed, developed and adapted two versions of a care bundle intervention, the SAFER Mental Health care bundle for adult and youth inpatient mental health settings (SAFER-MH and SAFER-YMH respectively) that look to address these concerns through the introduction of new or improved processes of care.

Methods and analysis

Two uncontrolled before-and-after feasibility studies, where all participants will receive the intervention. We will examine the feasibility and acceptability of the SAFER-MH in inpatient mental health settings in patients aged 18 years or older who are being discharged and the feasibility and acceptability of the SAFER-YMH intervention in inpatient mental health settings in patients aged between 14 to 18 years who are being discharged. The baseline period and intervention periods are both 6 weeks. SAFER-MH will be implemented in three wards and SAFER-YMH in one or two wards, ideally across different trusts within England. We will use quantitative (e.g. questionnaires, completion forms) and qualitative (e.g. interviews, process evaluation) methods to assess the acceptability and feasibility of the two versions of the intervention. The findings will inform whether a main effectiveness trial is feasible and, if so, how it should be designed, and how many patients/wards should be included.

Ethics and dissemination

Ethical approval was obtained from the National Health Service Cornwall and Plymouth Research Ethics Committee and Surrey Research Ethics Committee (reference: 22/SW/0096 and 22/LO/0404). Research findings will be disseminated with participating sites and shared in a various ways to engage different audiences. We will present findings at international and national conferences, publish in open-access, peer-reviewed journals.

Strengths and limitations of this study

- This is a novel best practice intervention for making discharge from acute inpatient mental health settings to the community safer and more patient-centred
- The study design, management and data analysis benefit from the input of experts by experience.
- An in-depth mixed method approach (qualitative and quantitative) will be used to evaluate the study and inform the feasibility and key outcomes of the main trial.
- A potential limitation of this study is the lack of a control condition, although an extensive baseline phase is applied for comparing data before and after introducing the intervention.

Keywords

Feasibility Study
Protocol
Mental Health

For peer review only - http://bmjopen.bmj.com/site/about/guidelines.xhtml

Discharge
Care Transitions

Word count 3486

Introduction

It is estimated that one in four adults and one in ten children will be affected by mental or neurological disorders at some point in their lives (1,2). Mental illness has become the single largest cause of disability in England, costing the NHS over £13.3 billion in 2019/20 alone and is rising among both children and adults (3–5). Individuals that are admitted to inpatient mental health services are at higher risk of adverse outcomes such as shorter life expectancy; increased risk for homelessness; self-harm, violence and criminal activity; and increased likelihood of many other adverse psychosocial outcomes, including loneliness, isolation and stigma (6–10).

Transitions into and out of acute mental health hospitals are risky stages in the health care pathway associated with patient safety issues. The absence of continuity of care is an important risk factor for patient safety incidents during transitions of care (8). Other patient safety risk factors may include; difficulties of medication management across the care system; delayed discharge often arising from insufficient housing, social or community care provision and, lack of information sharing between services (primarily between primary and secondary care)(6,11–14).

Multi-faceted interventions have been increasingly used to improve patient safety and reduce readmissions in complex care transitions (11,15–17) . The SAFER Patient Flow Bundle is an exemplar multifaceted intervention, developed by NHS England/Improvement to improve discharge from acute hospitals to the community. (18). The SAFER Patient Flow bundle consists of five components: 1) Senior review (before midday); 2) Expected discharge date and clinical criteria for discharge; 3) Early assessments to improve patient flow; 4) Early discharge (aiming to discharge patients before midday); and 5) Review a multi-disciplinary review for patients with increased lengths of stay (18). Many of the individual components of the SAFER bundle are used as standard practice or best practice guidelines in policy (19). Preliminary evidence obtained by case studies across the country is promising as it shows that SAFER has resulted in reduced length of patient stay in different hospitals, diminished discharge delays with minimal complications and decreased re-admissions or contact with primary care. Furthermore, patient and staff satisfaction have increased (20).

Although protocols, webinars, and/or other tools have been developed to facilitate the implementation of SAFER (20), there is currently lack of evidence examining its

For peer review only - http://bmjopen.bmj.com/site/about/guidelines.xhtml

appropriateness within a mental health setting. To address this gap, we conducted a series of co-design empirical studies with multiple stakeholders including patients, carers, professionals and academics (21,22). These led to the development of two adapted versions of the SAFER patient flow bundle tailored to mental health, one for an adult acute mental health hospital (SAFER-MH) and one for Youth (Child and Adolescent) Mental Health Services (SAFER-YMH). Now that the bundle has been developed, we aim to generate data regarding its feasibility and acceptability, which will enrich the evidence base about its future wider adoption in NHS services.

The primary objectives of this research are therefore to:

1. examine whether the SAFER-MH and SAFER-YMH bundles are feasible and acceptable (based on standardised quantitative measures, and qualitative data) to patients, carers and professionals,
2. identify specific principle(s) of SAFER-MH and SAFER-YMH which need to be adapted to make it more acceptable, and increase its prospect of long-term implementation
3. examine the adherence to the delivery of the intervention (measured using a purposefully designed self-report form and recruitment rates), completion rate and appropriateness of the outcome measures and recruitment strategies
4. explore whether healthcare professional and patient engagement with the SAFER-MH and SAFER-YMH interventions can be increased using evidence-based behavioural science techniques

Methods and Analysis

Study design

This study will include two concurrent distinct studies, receiving adapted versions of the same intervention:

- Phase 1: SAFER-Mental Health (MH) for adults and,
- Phase 2: SAFER-Youth Mental Health (YMH) for children and adolescents which includes an adaption of the original SAFER-MH for young people.

These will be uncontrolled before-and-after feasibility studies where all participants will receive the intervention. We will use quantitative (e.g. questionnaires, completion forms) and qualitative (e.g. interviews, process evaluation) methods to assess acceptability and feasibility.

Intervention

The SAFER-MH is a co-designed adapted version of the SAFER Patient Flow Bundle (NHS Improvement) intervention (20) and will be delivered to patients as part of their normal care pathway. The intervention was adapted based on 35 stakeholder interviews and RAND consensus methods with multi-disciplinary experts and professionals (21,22). SAFER-YMH involved a further level of co-design to ensure that the intervention is suitable for adolescent services. This involved presenting the adult version to professional and patients' groups to discuss relevant changes to be made to make it more appropriate for adolescent services. First, we aim to identify the elements of existing practice in the pilot sites before implementing the SAFER interventions, in both studies. The intervention focuses on promoting best practice guidance and has three key stages: Admission, Discharge and Weekly Tasks and are discussed below:

1. Admission

At admission, the intervention group will complete three key tasks: 1) Setting criteria for discharge and an estimated discharge day, 2) Identifying early social information that will help plan for discharge and, 3) Introducing the Patient Written Discharge Plan. This will be structured within two documents including the admissions social information capture

For peer review only - http://bmjopen.bmj.com/site/about/guidelines.xhtml

document and the transitions checklist, which will be completed at multiple times in the patient journey.

2. Weekly Tasks

There are three weekly tasks that will form part of this assessment 1) Senior review of discharge readiness, 2) Multi-disciplinary discharge team meeting and, 3) Multi-agency discharge team meeting.

3. Discharge

At discharge, there will be two key tasks 1) Co Producing a high-quality patient written discharge plan and, 2) Ensuring the patient has their copy of the patient written discharge plan.

Participants

All patients admitted to pilot wards during the study period (14 weeks from November 2022 onwards) will receive the intervention, as it is a best practice intervention delivered as a service improvement change in care. Each individual participant that is due to be discharged from the pilot ward will then be given the option to consent (parental/guardian consent for under 16s) whether to be a participant in the research studies by participating in the interviews and by completing relevant questionnaires. Patient participants will be adults (phase 1) or young people (phase 2) with mental illness who were admitted to an inpatient ward. Healthcare professionals that will be delivering the intervention and carers/family members of the patients (who consent to be involved) will also be involved in the interview components of the study (patient and carers interviewees can decide whether to be interviewed alone or together).

We will collect data from all consenting patients that are discharged during the baseline and intervention stages, irrespective of whether their inpatient stay began before this period. In the case of an unplanned or early discharge, we will still collect data from consenting patients that have received the intervention, if a patient is not discharged from the pilot ward (due to transfer etc.), they will be ineligible to participant.

Eligibility criteria

Eligible sites

Eligible sites will acute adult inpatient mental health wards. As this is a feasibility study will aim to test on a variety of wards (i.e., female, male or mixed).

Phase 1: SAFER-MH

The eligibility criteria for individual patient participants to take part in the SAFER-MH study reflects the eligibility criteria for services. We will include adult patients aged 18-65 years, informal carers for adult patients with mental health problems aged over 18, healthcare and professionals that are involved in delivering the intervention and informal carers of patients (for process evaluation). All patients will receive the service-level intervention but will choose to participate in the study if they feel they have the capacity consent.

Phase 2: SAFER-YMH

The eligibility criteria to take part in the feasibility study reflects the eligibility criteria for services. Any young person accessing inpatient CAMHS services would be eligible to participate, a parent/carer of a young person accessing inpatient CAMHS services and healthcare Professionals that are involved in delivering the intervention and parents/guardians of the children. All patients will receive the service-level intervention but

For peer review only - http://bmjopen.bmj.com/site/about/guidelines.xhtml

will choose to participate in the study if they (their parents/guardians) feel they have the capacity and consent.

Recruitment and Data Collection
Inpatient mental health ward sites

Existing networks will be used to identify eligible services (wards) for the SAFER-MH/YMH studies. The study team will meet with identified services (face-to-face or remotely) to fully explain the purposes of the studies and describe their requirements. Informed consent for services to participate will be provided by the research lead/authorised person in each service, acting as 'guardian' for patients in their care. This will follow agreement with each local ward team clarifying willingness to undertake the SAFER-MH/YMH interventions. Service consent to participate in the study will be formalised through written agreements. The number of services approached, declining, or considered not eligible will be recorded.

Phase 1: SAFER-MH We aim to recruit three pilot inpatient wards across different trusts in the north west of England to reflect variation of NHS services in England.

Phase 2: SAFER-YMH
We aim to recruit one or two pilot inpatient wards within one trust in the North-West of England. We do not aim to recruit additional trusts because due to funding restrictions, phase 2 must be completed by March 2023.

Patient Participants
All patients on a participating ward will receive the intervention as it is a service level change. However, we will identify patients that are willing to participate in the study assessments (questionnaires and interviews). A ward co-ordinator (e.g., nurse or other health professional) working within the participating services will be asked to co-ordinate handing out questionnaire packs to eligible patients. Potentially eligible patients will be assigned an individual study ID and given an invitation pack close to their expected discharge. This includes an Invitation letter, Patient Information Sheet (PIS), and a Consent to Contact form.

On receipt of an invitation pack, the potential participant will complete the included documents and return to the research practitioner on site. Consent for researchers to contact the potential participant regarding the study will be obtained, in line with the definition outlined in Article 4(11) of the GDPR guidance, "any freely given, specific, informed and unambiguous indication of the data subject's wishes by which he or she, by a statement or by a clear affirmative action, signifies agreement to the processing of personal data relating to him or her" (European Union, 2016). Consent to be contacted with a study recruitment pack will therefore be implied by completion and return of a completed Consent to Contact form.

Assent for young patient participants

Assent will be sought from children / young persons' parents/guardians, or others who are not legally empowered to give consent in phase 2.

Carer/parent/guardian Participants

A study pack will be handed to each patient at discharge, this will include a carer pack, which they can choose to pass on or discard. The pack will include details of the study, paper questionnaires and a paper consent to contact form for interviews, also an online link

For peer review only - http://bmjopen.bmj.com/site/about/guidelines.xhtml

to assess the documents remotely. For patients who agree to be contacted for an interview, we will ask again if they would like to share the information pack and contact details with their informal carer when contacted.

Professional Participants

Staff participants will be those working directly with patient participants on participating mental health wards (i.e. nurses, health care assistants and doctors). Staff will self-identify to be involved in interviews and questionnaires through posters on the ward and emails sent by the trust research clinician/gatekeeper containing the study information pack (PIS, invitation letter, consent to contact form). All staff that complete the training will be given the opportunity to complete the evaluation survey.

Procedure
Pre-intervention (baseline) phase

This phase will consist of the usual care that the patients in the inpatient wards receive and will last for 6 weeks (see Table 1).. We will assess this current practice by conducting interviews and by inspecting current documentation sheets. These will be completed by one on-site research practitioner. We will perform individual online interviews with the patients, mental health professionals, and carers (relatives) to assess the degree of the satisfaction of the patients and carers as well as their views/thoughts regarding their current discharge plans. At the end of this phase, patients will be asked to complete a series of study questionnaires, including the EQUIP PROM scale (23), which measures the involvement of both the patients and carers in health care planning during/after discharge, The Care Transitions Measure (CTM) (24), and a five-item patient-centred scale focusing on mental health discharge outcomes that were identified as important to patients in previous work (9). Patients, carers, and professionals will also be invited to be interviewed about their experiences of usual care, via the study information pack.

Table 1: Data collection procedures

Patient/Carer Outcome Measures (n=45):<br>- EQUIP-PROM scale, and,<br>- 5-items regarding general satisfaction about discharge planning		Patient/Carer Outcome Measures (n=45):<br>• EQUIP-PROM scale,<br>• 5-items regarding general satisfaction, and<br>• the Care Transitions Measure (CTM)<br><br>Staff Outcome Measures (n=45):<br>• AIM<br>• IAM<br>• FIM<br>• Adherence to the delivery of the Safer patient flow bundle will be measured<br>• using self-report questionnaires

For peer review only - http://bmjopen.bmj.com/site/about/guidelines.xhtml

Training phase

The training will be a series of self-led short videos and self-help manuals to the pilot ward staff participating in the study. This phase will last for 2 weeks. We aim to use evaluation questionnaires following the training. Interviews in the training phase will explore whether the interventions have been fully comprehended by staff and ensure that we have enough time to solve any problems regarding the training as they arise.

Intervention phase

This phase will last for 6 weeks during which the wards will receive the SAFER-MH and SAFER-YMH patient flow bundle (figure 1). Data regarding the adherence of the mental health professionals to using the SAFER-MH bundle will be collected. Individual online interviews (Zoom) will be conducted with patients and carers (within three months after discharge), and with mental health professionals (within one month after the completion of the 6-week intervention phase) to assess the feasibility and acceptability of the SAFER–MH bundle. Each patient/carer will only be interviewed once, professionals may be interviewed more than once in different stages of the project. At the end of this 6-week phase, patients will be asked to complete the same study scales that were administered during the baseline and which were completed by a different pool of patient participants discharged at the time. We will also administer the three staff outcome measures after the intervention phase is complete.

Figure 1: Timeline of events

Outcomes

The aim of the evaluation will be to determine the feasibility and acceptability of the SAFER-MH and SAFER-YMH interventions by patients, carers and professionals. Additionally, we aim to identify the completion rates and if there are any changes in the key outcomes. As this is a pre-post uncontrolled feasibility study, it will only give us a first indication about the appropriateness of the outcome measures whereas we will primarily focus on whether the SAFER-MH and SAFER-YMH is acceptable and, if so, for whom. We chose 6 week phases, as studies in UK settings indicate average adult length of stays are around 22-36 days and The NHS Mental Health Implementation Plan aims to reduce length of inpatient psychiatric stays to a maximum of 32 days, enabling us to capture a considerable number of patients during the study(27,28).

Quantitative Outcomes

These will be measured in the baseline phase and intervention phase with different patients. To assess patients and carers perspectives, the quantitative outcomes will be measured using paper/online versions of a series of questionnaires which include: 1) the EQUIP patient reported outcome measure, a 14-item co-designed measure to assess patient/carer views of care planning in mental health (23); 2) a short 4-item version of the Care Transitions Measure

For peer review only - http://bmjopen.bmj.com/site/about/guidelines.xhtml

(CTM-4) (24), and 3) the five novel items assessing the safety of mental health care transitions (9), properties of these outcome measures can be found in table 2.

To assess professional perspectives, we will use three measures the Acceptability, Appropriateness and Feasibility of Intervention Measure (AIM, IAM and FIM). Each of the three measure contains 4-items to assess professionals' perspectives of a new intervention. These measures will be administered after the intervention phase only (not the baseline). Adherence to the delivery of the SAFER-MH and SAFER-YMH bundle will be measured using a purposefully designed self-report form and recruitment rates will be recorded at each stage.

Table 2: Properties of outcome measures used

Validated Outcome Measure	Psychometric Properties	Scoring system
EQUIP	Scale has acceptable scalability (Ho = .69), reliability (alpha = .92), fit to the Rasch model ($\chi 2(70) = 97.25$, $p = .02$), and no differential item functioning or locally dependent items.	It uses a 5-point Likert scale ranging from 1= 'strongly disagree' to 5 = 'strongly agree', with a middle neutral value meaning "neither agree nor disagree." Higher scores indicate higher-perceived quality care planning.
CTM-4	The Cronbach's alpha for CTM-4 is 0.85.	The CTM items are rated on a four-point Likert scale ranging from 1 = 'strongly disagree' to 4 = 'strongly agree', with a fifth response being 'don't know/don't remember/not applicable'. The summarised score (excluding the 'not applicable' scores) is computed as the total sum divided with the number of answered items, minus 1 and multiplied by 100 to get a total score (0–100) for each respondent. Higher scores indicate better patient/carer experience of care transitions
AIM, FIM, IAM	The alphas for 5-item scales were between 0.87 and 0.89. Scale refinement based on measure-specific CFAs and Cronbach alphas using vignette data produced 4-item scales (α's from 0.85 to 0.91). A three-factor CFA exhibited acceptable fit (CFI = 0.96, RMSEA = 0.08) and high factor loadings (0.75 to 0.89), indicating structural validity. ANOVA showed significant main effects, indicating known-groups validity. Test-retest reliability coefficients ranged from 0.73 to 0.88.	1 = Completely disagree, 2 = Disagree, 3 = Neither agree nor disagree, 4 = Agree, 5 =Completely agree Scoring Instructions: Scales can be created for each measure by averaging responses. Scale values range from 1 to 5. Higher scores indicate higher acceptability, appropriateness, and feasibility of the intervention.

In-depth Qualitative Outcome data

For peer review only - http://bmjopen.bmj.com/site/about/guidelines.xhtml

A qualitative process evaluation will be conducted using semi-structured interviews to provide understanding of the study delivery and its potential scale-up. All participants that complete questionnaires will be given the opportunity to volunteer to be interviewed. We aim to interview patients and carers at baseline and intervention, whereas professionals will be interviewed at all three stages (baseline, training, and intervention). All participants (including children) will be remunerated in vouchers for participation.

Data Analysis Plan

We will be following the published extension to Consolidated Standards of Reporting Trials (CONSORT) (29) when reporting the results of this feasibility study (26) . As this is a feasibility study, no formal sample size calculation has been conducted. Our targeted sample size (n=90, phase 1, n=40, phase 2) should be large enough to consider the practicalities of recruitment and delivering the interventions.
Descriptive statistics for intervention feasibility, acceptability and appropriateness will be presented. A chart of participant flow during the whole study will be plotted (CONSORT flow chart) which will include information on the retention/withdrawal (including timing and reasons) of wards during the evaluation. We will also present the number of health practitioners who completed the questionnaires and interviews against the number of professionals working in the wards during the evaluation, and the number of patients/carers completing the questionnaires/interviews against those discharged during the duration of the study. We will also conduct basic psychometric analyses to verify the scale structure and internal consistency of the used questionnaires. For explorative purposes, we will examine differences between the three measures completed by patients recruited during the baseline phase and the measures completed by the patients during the intervention phase. Before starting with the analyses, we will examine if the data is normally distributed. If the data are normally distributed, parametric tests will be used, otherwise non-parametric tests will be used. The significance level for all analyses will be $p \leq 0.05$. The data analyst will be blinded to the phase allocation (baseline or intervention phase).

Qualitative Analysis

We will use a framework approach to qualitative analysis, to inductively code and organise the data and identify emerging themes from the interviews. The frameworks will be based on two underpinning theoretical frameworks commonly used in similar research: the Conceptual Framework for Implementation Fidelity (5 domains), Theoretical Framework of Acceptability (31) and the Theoretical Domains Framework (14 domains). The coding process will follow these steps:

1. Familiarisation (researchers emerging themselves in the data),

2. Identifying a thematic framework (to generate an index of data based on a priori questions and issues raised by participants),

3. Indexing (applying thematic framework to the data using NVivo software),

4. Charting (Rearranging data in line with thematic framework), and

5. Mapping and interpretation (Using the charts to define concepts, map the range and nature of phenomena and find associations between themes).

Integration of quantitative and qualitative data

For peer review only - http://bmjopen.bmj.com/site/about/guidelines.xhtml

The quantitative and qualitative results will be initially considered separately but we aim to examine how they converge or differ when presenting the overall conclusions (28). Furthermore, the qualitative data collected across the two studies will be used to help explain or elaborate on the quantitative data (28).

Patient and public involvement

This study has a patient and public involvement service-user co-researcher who has been involved in the design and delivery of the study (AG). Patient and public involvement has been integral to this study and the development of the intervention which has been based on a series of stakeholder consultations, workshops and consensus methods (9,10,21,23). A core group of five patient and public involvement representatives form a steering committee for this study.

Ethics & Dissemination

Ethical approval was obtained from the National Health Service Cornwall and Plymouth Research Ethics Committee and Surrey Research Ethics Committee (reference: 22/SW/0096 and 22/LO/0404, respectively).

The study findings will be shared with participating inpatient sites in a range of ways to engage different audiences, as well as via presentations at national and international conferences and publishing in open-access peer-reviewed journals. Findings will be presented at a workshop for patients and carers a vlog will be created.

Discussion

This study aims to understand how to improve information sharing and capture between services during patient discharge from mental health hospitals to community services. Extensive stakeholder engagement with patients, carers, mental health professionals and academics has informed the intervention refinement and study co-design. We propose a pragmatic approach to test the feasibility and acceptability of a novel intervention which will add value to the evidence base about how information sharing/capture and patient/carer knowledge and involvement around discharge improves patient safety, health care utilisation and other important patient outcomes.

The findings will have to be interpreted and evaluated considering the limitations of the study design. For example, this is a pre-post uncontrolled study and the potential influence of inpatient settings cannot be controlled for through randomisation. Another limitation is that the interventions have only been developed in English and future work will need to explore how to culturally adapt it. Furthermore, some elements of the intervention may not be suitable for individuals with dementia who often occupy mental health inpatient beds (29), however funding has been obtained to develop this in future work. Finally, he intervention sites will be identified through collaborators, so selection bias must be considered.

The feasibility study will include paper and pdf editable versions of the intervention documents (e.g. social information capture and Patient Written Discharge Plan) which have not been integrated into the Trusts' electronic records yet. Understanding how the intervention documents will fit within the Trusts' electronic records in a key learning task of this feasibility study which will potentially inform a the larger RCT. Similarly, as this is a feasibility study, a we are mostly interested in the completion rate of the scales, and the views by patients/carers and staff members about the relevance of these measures to their experiences in relation to hospital discharge. We wont have enough power to generate any meaningful quantitative analysis using our outcome measures and we wontcollect quantitative data around

readmission rates or adverse events at this stage However, if this study produces convincing evidence that the intervention is feasible/acceptable we will request further funding to conduct a an RCT which will involve more extensive data collection (including routinely collected data on readmissions and adverse events) and a comprehensive quantitative analysis plan a definitive RCT. There is considerable evidence about the importance of community follow-up of patients at post-discharge, Although this is a pre-discharge intervention, there is growing evidence that continuity of care and post-discharge follow-up of patients could enhance safety. Depending on the feedback that we will obtain by the intervention recipients/deliverers during this feasibility study, we will explore whether further adaptations are needed to include post-discharge elements in the intervention protocol.

Improving safety at mental health discharge is a key concern for patients, professionals and policy makers. Therefore, interventions aiming to improve knowledge sharing, shared decision-making and involvement of patients/carers are considered preferable (10,23,30). This study will determine the feasibility and acceptability of a multicomponent intervention to improve involvement of patients and/or carers in discharge planning. This will provide evidence for the acceptability of the SAFER-MH and SAFER-YMH to inform future improvements to scale to a randomised controlled trial, with concurrent process evaluation and economic evaluation.

Authors' contributions: NT and MP had the initial research idea and obtained funding for this study. NT, MP, and IA formulated the research questions and designed the study. AH NT, IA and MP devised the qualitative and statistical analysis plan. NT, MP and IA drafted the protocol. AG led the patient and public involvement components of the study design. JL drafted the protocol and completed ethics documentation/application. RK, CP, SG, NK, CJA, TB, SC, CR substantially contributed to the manuscript by providing review comments and edits and approved the final manuscript.

Funding statement: This work is supported by the NIHR Greater Manchester Patient Safety Translational Research Centre (grant number: PSTRC-2016-003) and NIHR School for Primary Care Research Capacity award 21/22 (grant number C015)Postdoctoral fellowship award. The work. The views expressed are those of the author(s) and not necessarily those of the
NIHR or the Department of Health and Social Care.

Competing interests statement. None

Acknowledgements

We would like to thank all of the patient, public, carer and professional stakeholders who have been involved in the co-design and development of the SAFER-MH intervention to date.

References

1. Mcmanus S, Meltzer H, Brugha T, Bebbington P, Jenkins R. Adult psychiatric

For peer review only - http://bmjopen.bmj.com/site/about/guidelines.xhtml

morbidity in England , 2007 Results of a household survey. Appendices and Glossary [Internet]. Health San Francisco. 2007 [cited 2022 Jul 25]. Available from: https://digital.nhs.uk/data-and-information/publications/statistical/adult-psychiatric-morbidity-survey/adult-psychiatric-morbidity-in-england-2007-results-of-a-household-survey

2. NHS England » Mental health [Internet]. [cited 2022 Aug 3]. Available from: https://www.england.nhs.uk/mental-health/
3. Ford T, John A, bmj DG-, 2021 undefined. Mental health of children and young people during pandemic. bmj.com [Internet]. 2021 Mar 10 [cited 2022 Jul 25];372. Available from: https://www.bmj.com/content/372/bmj.n614.full
4. Newlove-Delgado T, McManus S, Sadler K, Thandi S, Vizard T, Cartwright C, et al. Child mental health in England before and during the COVID-19 lockdown. The Lancet Psychiatry. 2021 May 1;8(5):353–4.
5. British Medical Association. The impact of the pandemic on population health and health inequalities BMA Covid Review 5.
6. Forchuk C, Russell G, Kingston-Macclure S, Turner K, Dill S. From psychiatric ward to the streets and shelters. Bombardieri Diaz, Double, Draine, Drake, Drake, Goering, Jones, Koegel, Krupa, Kuno, Lamb, Lamb, Leff, McChesney, Olfson, Philp, Ravignat, Robertson, Rosenheck, Stuart, Sullivan, Susser, Wong C, editor. J Psychiatr Ment Health Nurs [Internet]. 2006;13(3):301–8. Available from: http://ovidsp.ovid.com/ovidweb.cgi?T=JS&PAGE=reference&D=psyc5&NEWS=N&AN=2006-07123-007
7. Walter F, Carr MJ, Mok PLH, Antonsen S, Pedersen CB, Appleby L, et al. Multiple adverse outcomes following first discharge from inpatient psychiatric care: a national cohort study. The Lancet Psychiatry. 2019 Jul 1;6(7):582–9.
8. Healthcare Quality Improvement Partnership. National Confidential Inquiry into Suicide and Safety in Mental Health. ANNUAL REPORT: ENGLAND, NORTHERN IRELAND, SCOTLAND AND WALES 2021 [Internet]. 2021 [cited 2021 Jul 14]. Available from: https://www.research.manchester.ac.uk/portal/en/projects/national-confidential-inquiry-into-suicide-and-safety-in-mental-health(788f9475-cadb-4697-bb82-817638044b7b).html
9. Tyler N, Wright N, Grundy A, Waring J. Developing a core outcome set for interventions to improve discharge from mental health inpatient services: A survey, Delphi and consensus meeting with key stakeholder groups. BMJ Open. 2020.
10. Tyler N, Wright N, Panagioti M, Grundy A, Waring J. What does safety in mental health care transitions mean for service users and other stakeholder groups: An open-ended questionnaire study. Heal Expect. 2020;
11. Tyler N, Wright N, Waring J. Interventions to improve discharge from acute adult mental health inpatient care to the community: systematic review and narrative synthesis. BMC Health Serv Res [Internet]. 2019 Dec 25 [cited 2019 Dec 12];19(1):883. Available from: https://bmchealthservres.biomedcentral.com/articles/10.1186/s12913-019-4658-0
12. Steffen S, Kösters M, Becker T, Puschner B. Discharge planning in mental health care: A systematic review of the recent literature. Acta Psychiatrica Scandinavica. 2009.
13. Bonsack C, Golay P, Manetti SG, Gebel S, Ferrari P, Besse C, et al. Linking primary and secondary care after psychiatric hospitalization: Comparison between transitional case management setting and routine care for common mental disorders. Bonsack Bonsack, Boyer, Derogatis, Dixon, Goldman, Guelfi, Heggestad, Henderson, Hengartner, Juven-Wetzler, Klinkenberg, Lamb, Nelson, Olfson, Pomini, Puschner, Qin, Steffen, Tomita, Vasiliadis, Vigod, Zusman B, editor. Front Psychiatry [Internet]. 2016;7. Available from: http://ovidsp.ovid.com/ovidweb.cgi?T=JS&PAGE=reference&D=psyc13a&NEWS=N&AN=2016-29356-001
14. Keers RN, Williams SD, Vattakatuchery JJ, Brown P, Miller J, Prescott L, et al.

Medication safety at the interface: evaluating risks associated with discharge prescriptions from mental health hospitals. J Clin Pharm Ther [Internet]. 2015 Dec 1 [cited 2022 Jul 25];40(6):645–54. Available from: https://pubmed.ncbi.nlm.nih.gov/26534824/
15. Tyler N, Wright N, Panagioti M, Grundy A, Waring J. What does safety in mental healthcare transitions mean for service users and other stakeholder groups: An open-ended questionnaire study. Heal Expect. 2021 May 1;24(S1):185–94.
16. Gonçalves-Bradley DC, Lannin NA, Clemson L, Cameron ID, Shepperd S. Discharge planning from hospital. Cochrane Database Syst Rev [Internet]. 2022 Feb 24 [cited 2022 Jul 19];2022(2). Available from: https://www.cochranelibrary.com/cdsr/doi/10.1002/14651858.CD000313.pub6/full
17. Coffey A, Leahy-Warren P, Savage E, Hegarty J, Cornally N, Day MR, et al. Interventions to Promote Early Discharge and Avoid Inappropriate Hospital (Re)Admission: A Systematic Review. Int J Environ Res Public Health [Internet]. 2019 Jul 2 [cited 2022 Mar 29];16(14). Available from: https://pubmed.ncbi.nlm.nih.gov/31295933/
18. Steffen S, Kösters M, Becker T, Puschner B. Discharge planning in mental health care: A systematic review of the recent literature. Acta Psychiatr Scand [Internet]. 2009;120(1):1–9. Available from: https://www.scopus.com/inward/record.uri?eid=2-s2.0-66749126657&doi=10.1111%2Fj.1600-0447.2009.01373.x&partnerID=40&md5=85f563fd242fbfaf95f762a69e7231fe
19. Leppin AL, Gionfriddo MR, Kessler M, Brito JP, Mair FS, Gallacher K, et al. Preventing 30-Day Hospital Readmissions: A Systematic Review and Meta-analysis of Randomized Trials. JAMA Intern Med [Internet]. 2014 Jul 1 [cited 2022 Jul 19];174(7):1095–107. Available from: https://jamanetwork.com/journals/jamainternalmedicine/fullarticle/1868538
20. NHS Improvement. Rapid improvement guide: the SAFER patient flow bundle [Internet]. 2016 [cited 2019 Nov 12]. Available from: https://improvement.nhs.uk/resources/rapid-improvement-guide-safer-patient-flow-bundle/
21. National Institute for Health and Care Excellence. Transition between inpatient mental health settings and community or care home settings | Guidance and guidelines | NICE [Internet]. NICE; 2016 [cited 2018 Dec 20]. Available from: https://www.nice.org.uk/guidance/ng53
22. The SAFER Patient Flow Bundle | Fab NHS Stuff [Internet]. [cited 2022 Aug 22]. Available from: https://fabnhsstuff.net/fab-stuff/28574-the-safer-patient-flow-bundle
23. Tyler N, Planner C, Byrne M, Blakeman T, Keers RN, Wright O, et al. Developing Best Practice Guidance for Discharge Planning Using the RAND/UCLA Appropriateness Method. Front Psychiatry. 2021 Dec 3;12:2204.
24. Tyler, Natasha; Daker-White, Gavin; Grundy, Andrew; Quinliivan, Leah; Armitage, Chris J.; Campbell, Stephen; Panagioti, Maria. The effects of the Covid-19 lockdown on quality and safety in mental health care transitions in England (In Press). BJPsych Open. 2021;
25. Bee P, Gibbons C, Callaghan P, Fraser C, Lovell K. Evaluating and Quantifying User and Carer Involvement in Mental Health Care Planning (EQUIP): Co-Development of a New Patient-Reported Outcome Measure. PLoS One [Internet]. 2016 Mar 1 [cited 2022 Jul 21];11(3):e0149973. Available from: https://journals.plos.org/plosone/article?id=10.1371/journal.pone.0149973
26. Flink M, Tessma M, Småstuen MC, Lindblad M, Coleman EA, Ekstedt M. Measuring care transitions in Sweden: validation of the care transitions measure. Int J Qual Heal Care [Internet]. 2018 May 1 [cited 2022 Jul 21];30(4):291. Available from: /pmc/articles/PMC5928451/
27. National Health Service. NHS Mental Health Implementation Plan 2019/20 – 2023/24. 2019;
28. Crossley N, Sweeney B. Patient and service-level factors affecting length of inpatient

For peer review only - http://bmjopen.bmj.com/site/about/guidelines.xhtml

stay in an acute mental health service: A retrospective case cohort study. BMC Psychiatry [Internet]. 2020 Sep 7 [cited 2023 Jan 18];20(1):1–9. Available from: https://bmcpsychiatry.biomedcentral.com/articles/10.1186/s12888-020-02846-z
29. Saint-Raymond A, Hill S, Martines J, Bahl R, Fontaine O, Bero L. Consort 2010. The Lancet. 2010.
30. Lancaster GA, Thabane L. Guidelines for reporting non-randomised pilot and feasibility studies. Pilot Feasibility Stud [Internet]. 2019 Oct 6 [cited 2022 Aug 22];5(1):1–6. Available from: https://pilotfeasibilitystudies.biomedcentral.com/articles/10.1186/s40814-019-0499-1
31. Sekhon M, Cartwright M, Francis JJ. Acceptability of healthcare interventions: an overview of reviews and development of a theoretical framework. BMC Heal Serv Res 2017 171 [Internet]. 2017 Jan 26 [cited 2021 Aug 10];17(1):1–13. Available from: https://bmchealthservres.biomedcentral.com/articles/10.1186/s12913-017-2031-8
32. Creswell JW. A Concise Introduction to Mixed Methods Research [Internet]. SAGE Publications; 2014 [cited 2016 Mar 3]. 152 p. Available from: https://books.google.com/books?hl=en&lr=&id=51UXBAAAQBAJ&pgis=1
33. Edmans BG, Wolverson E, Dunning R, Slann M, Russell G, Crowther G, et al. Inpatient psychiatric care for patients with dementia at four sites in the United Kingdom. Int J Geriatr Psychiatry [Internet]. 2022 Feb 1 [cited 2022 Jul 25];37(2). Available from: https://onlinelibrary.wiley.com/doi/full/10.1002/gps.5658
34. Slade M. Implementing shared decision making in routine mental health care. World Psychiatry [Internet]. 2017 Jun 1 [cited 2021 Nov 4];16(2):146. Available from: /pmc/articles/PMC5428178/

For peer review only

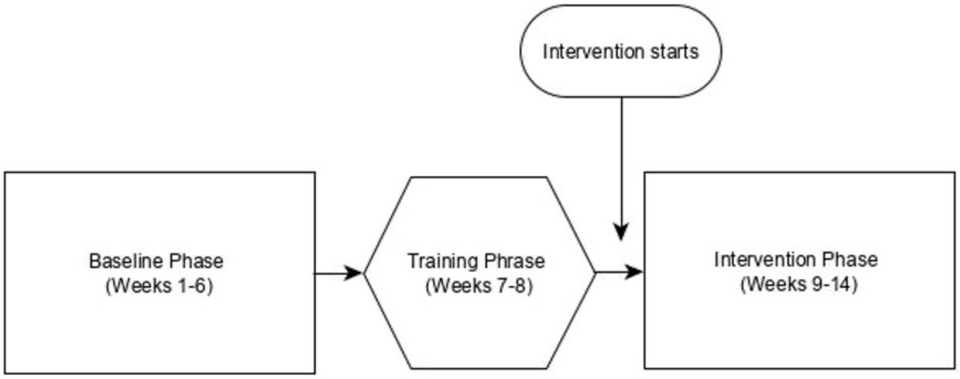

Figure 1: Timeline of events

159x66mm (144 x 144 DPI)

Protocol for a non-randomised feasibility study evaluating a co-designed care bundle to improve patient safety at discharge from adult and adolescent mental health services (SAFER-MH and SAFER-YMH)

Journal:	*BMJ Open*
Manuscript ID	bmjopen-2022-069216.R2
Article Type:	Protocol
Date Submitted by the Author:	22-Feb-2023
Complete List of Authors:	Tyler, Natasha; NIHR Greater Manchester Patient Safety Translational Research Centre; The University of Manchester, NIHR School for Primary Care Research<br>Angelakis, Ioannis; University of Liverpool, Department of Primary Care and Mental Health<br>Keers, Richard; The University of Manchester, Centre for Pharmacoepidemiology and Drug Safety Research<br>Planner, Claire; The University of Manchester, NIHR Greater Manchester Patient Safety Translational Research<br>hodkinson, alexander; University of Manchester, Centre for Primary Care<br>Giles, Sally; University of Manchester, NIHR Greater Manchester Primary Care Patient Safety Translational Research Centre<br>Grundy, Andrew; The University of Manchester<br>Kapur, Navneet; The University of Manchester, Centre for suicide prevention<br>Armitage, Chris; The University of Manchester, Manchester Centre for Health Psychology; The University of Manchester, NIHR Greater Manchester Patient Safety Translational Research Centre<br>Blakeman, Tom; The University of Manchester, NIHR Patient Safety Translational Research Centre; The University of Manchester, NIHR School for Primary Care Research<br>Campbell, Stephen; The University of Manchester, NIHR Greater Manchester Patient Safety Translational Research Centre<br>Robinson, Catherine; The University of Manchester<br>Leather, Jessica; The University of Manchester, NIHR Greater Manchester Patient Safety Translational Research Centre<br>Panagioti, Maria; The University of Manchester, NIHR Greater Manchester Patient Safety Translational Research Centre; The University of Manchester, NIHR School for Primary Care Research
**Primary Subject Heading**:	Mental health
Secondary Subject Heading:	Evidence based practice
Keywords:	MENTAL HEALTH, Child & adolescent psychiatry < PSYCHIATRY, Adult

	psychiatry < PSYCHIATRY

SCHOLARONE™
Manuscripts

Protocol for a non-randomised feasibility study evaluating a co-designed care bundle to improve patient safety at discharge from adult and adolescent mental health services (SAFER-MH and SAFER-YMH)

Tyler, N.,[1,2,6]* Angelakis, I.,[3] Keers, R.N.,[4,5] Planner, C.,[1,2] Hodkinson, A.,[6] Giles, S.,[1,2] Grundy, A. [7], Kapur, N. [1,8,9], Armitage, C.J.A [1,10,11], Blakeman, T.,[1,2], Campbell, S. [2], Robinson, C. [12], Leather, J. [1,10], Panagioti, M.[1,2,6]

1. National Institute for Health and Care Research Greater Manchester Patient Safety Translational Research Centre, Division of Population Health, Health Services Research and Primary Care, University of Manchester, UK
2. Centre for Primary Care Research, School for Primary Care Research, Division of Population Health, Health Services Research & Primary Care, University of Manchester, Manchester, UK
3. Institute of Population Health, Department of Primary Care and Mental Health, University of Liverpool, UK
4. Centre for Pharmacoepidemiology and Drug Safety, Division of Pharmacy and Optometry, University of Manchester, GM PSTRC
5. Suicide, Risk and Safety Research Unit, Greater Manchester Mental Health NHS Foundation Trust, Manchester
6. National Institute for Health and Care Research (NIHR) School for Primary Care Research, Division of Population Health, Health Services Research and Primary Care, School of Health Sciences, Faculty of Biology, Medicine and Health, Manchester Academic HealthScience Centre, University of Manchester, Manchester, UK
7. Division of Nursing, Midwifery & Social Work, School of Health Sciences, Faculty of Biology, Medicine, and Health, The University of Manchester
8. Centre for Mental Health & Safety, Division of Psychology & Mental Health, School of Health Sciences, Faculty of Biology, Medicine, and Health, The University of Manchester and Manchester Academic Health Sciences Centre, Manchester, UK
9. Greater Manchester Mental Health NHS Foundation Trust, Manchester, UK
10. Manchester Centre for Health Psychology, School of Health Sciences, University of Manchester, Manchester, UK
11. Manchester Academic Health Science Centre, Manchester University NHS Foundation Trust, Manchester, UK
12. Faculty of Biology, Medicine and Health, School of Health Sciences, University of Manchester, Manchester, UK

***Corresponding Author**
Dr. Natasha Tyler
Email address: natasha.tyler@manchester.ac.uk

For peer review only - http://bmjopen.bmj.com/site/about/guidelines.xhtml

Abstract

Introduction

Patients being discharged from inpatient mental wards often describe safety risks in terms of inadequate information sharing and involvement in discharge decisions. Through stakeholder engagement we co-designed, developed and adapted two versions of a care bundle intervention, the SAFER Mental Health care bundle for adult and youth inpatient mental health settings (SAFER-MH and SAFER-YMH respectively) that look to address these concerns through the introduction of new or improved processes of care.

Methods and analysis

Two uncontrolled before-and-after feasibility studies, where all participants will receive the intervention. We will examine the feasibility and acceptability of the SAFER-MH in inpatient mental health settings in patients aged 18 years or older who are being discharged and the feasibility and acceptability of the SAFER-YMH intervention in inpatient mental health settings in patients aged between 14 to 18 years who are being discharged. The baseline period and intervention periods are both 6 weeks. SAFER-MH will be implemented in three wards and SAFER-YMH in one or two wards, ideally across different trusts within England. We will use quantitative (e.g. questionnaires, completion forms) and qualitative (e.g. interviews, process evaluation) methods to assess the acceptability and feasibility of the two versions of the intervention. The findings will inform whether a main effectiveness trial is feasible and, if so, how it should be designed, and how many patients/wards should be included.

Ethics and dissemination

Ethical approval was obtained from the National Health Service Cornwall and Plymouth Research Ethics Committee and Surrey Research Ethics Committee (reference: 22/SW/0096 and 22/LO/0404). Research findings will be disseminated with participating sites and shared in a various ways to engage different audiences. We will present findings at international and national conferences, publish in open-access, peer-reviewed journals.

Strengths and limitations of this study

- This is a best practice intervention for making discharge from acute inpatient mental health settings to the community safer and more patient-centred
- The study design, management and data analysis benefit from the input of experts by experience.
- An in-depth mixed method approach (qualitative and quantitative) will be used to evaluate the study and inform the feasibility and key outcomes of the main trial.
- A potential limitation of this study is the lack of a control condition, although an extensive baseline phase is applied for comparing data before and after introducing the intervention.

Keywords

Feasibility Study
Protocol
Mental Health

For peer review only - http://bmjopen.bmj.com/site/about/guidelines.xhtml

Discharge
Care Transitions

Word count 3486

Introduction

It is estimated that one in four adults and one in ten children will be affected by mental or neurological disorders at some point in their lives (1,2). Mental illness has become the single largest cause of disability in England, costing the NHS over £13.3 billion in 2019/20 alone and is rising among both children and adults (3–5). Individuals that are admitted to inpatient mental health services are at higher risk of adverse outcomes such as shorter life expectancy; increased risk for homelessness; self-harm, violence and criminal activity; and increased likelihood of many other adverse psychosocial outcomes, including loneliness, isolation and stigma (6–10).

Transitions into and out of acute mental health hospitals are risky stages in the health care pathway associated with patient safety issues. The absence of continuity of care is an important risk factor for patient safety incidents during transitions of care (8). Other patient safety risk factors may include; difficulties of medication management across the care system; delayed discharge often arising from insufficient housing, social or community care provision and, lack of information sharing between services (primarily between primary and secondary care (6,11–15).

Multi-faceted interventions have been increasingly used to improve patient safety and reduce readmissions in complex care transitions (11,16–19) . The SAFER Patient Flow Bundle is an exemplar multifaceted intervention, developed by NHS England/Improvement to improve discharge from acute hospitals to the community (20). The SAFER Patient Flow bundle consists of five components: 1) Senior review (before midday); 2) Expected discharge date and clinical criteria for discharge; 3) Early assessments to improve patient flow; 4) Early discharge (aiming to discharge patients before midday); and 5) Review a multi-disciplinary review for patients with increased lengths of stay (20). Many of the individual components of the SAFER bundle are used as standard practice or best practice guidelines in policy (21). Preliminary evidence obtained by case studies across the country is promising as it shows that SAFER has resulted in reduced length of patient stay in different hospitals, diminished discharge delays with minimal complications and decreased re-admissions or contact with primary care. Furthermore, patient and staff satisfaction have increased (22).

Although protocols, webinars, and/or other tools have been developed to facilitate the implementation of SAFER (22), there is currently lack of evidence examining its

For peer review only - http://bmjopen.bmj.com/site/about/guidelines.xhtml

appropriateness within a mental health setting. To address this gap, we conducted a series of co-design empirical studies with multiple stakeholders including patients, carers, professionals and academics (23,24). These led to the development of two adapted versions of the SAFER patient flow bundle tailored to mental health, one for an adult acute mental health hospital (SAFER-MH) and one for Youth (Child and Adolescent) Mental Health Services (SAFER-YMH). Now that the bundle has been developed, we aim to generate data regarding its feasibility and acceptability, which will enrich the evidence base about its future wider adoption in NHS services.

The primary objectives of this research are therefore to:

1. examine whether the SAFER-MH and SAFER-YMH bundles are feasible and acceptable (based on standardised quantitative measures, and qualitative data) to patients, carers and professionals,
2. identify specific principle(s) of SAFER-MH and SAFER-YMH which need to be adapted to make it more acceptable, and increase its prospect of long-term implementation
3. examine the adherence to the delivery of the intervention (measured using a purposefully designed self-report form and recruitment rates), completion rate and appropriateness of the outcome measures and recruitment strategies
4. explore whether healthcare professional and patient engagement with the SAFER-MH and SAFER-YMH interventions can be increased using evidence-based behavioural science techniques

Methods and Analysis

Study design

This study will include two concurrent distinct studies, receiving adapted versions of the same intervention:

- Phase 1: SAFER-Mental Health (MH) for adults and,
- Phase 2: SAFER-Youth Mental Health (YMH) for children and adolescents which includes an adaption of the original SAFER-MH for young people.

These will be uncontrolled before-and-after feasibility studies where all participants will receive the intervention. We will use quantitative (e.g. questionnaires, completion forms) and qualitative (e.g. interviews, process evaluation) methods to assess acceptability and feasibility.

Intervention

The SAFER-MH is a co-designed adapted version of the SAFER Patient Flow Bundle (NHS Improvement) intervention (20) and will be delivered to patients as part of their normal care pathway. The intervention was adapted based on 35 stakeholder interviews and RAND consensus methods with multi-disciplinary experts and professionals (23,24). SAFER-YMH involved a further level of co-design to ensure that the intervention is suitable for adolescent services. This involved presenting the adult version to professional and patients' groups to discuss relevant changes to be made to make it more appropriate for adolescent services. First, we aim to identify the elements of existing practice in the pilot sites before implementing the SAFER interventions, in both studies. The intervention focuses on promoting best practice guidance and has three key stages: Admission, Discharge and Weekly Tasks and are discussed below:

1. Admission

At admission, the intervention group will complete three key tasks: 1) Setting criteria for discharge and an estimated discharge day, 2) Identifying early social information that will help plan for discharge and, 3) Introducing the Patient Written Discharge Plan. This will be structured within two documents including the admissions social information capture

document and the transitions checklist, which will be completed at multiple times in the patient journey.

2. Weekly Tasks
There are three weekly tasks that will form part of this assessment 1) Senior review of discharge readiness, 2) Multi-disciplinary discharge team meeting and, 3) Multi-agency discharge team meeting.

3. Discharge
At discharge, there will be two key tasks 1) Co Producing a high-quality patient written discharge plan and, 2) Ensuring the patient has their copy of the patient written discharge plan.

Participants

All patients admitted to pilot wards during the study period (14 weeks from November 2022 onwards) will receive the intervention, as it is a best practice intervention delivered as a service improvement change in care. Each individual participant that is due to be discharged from the pilot ward will then be given the option to consent (parental/guardian consent for under 16s) whether to be a participant in the research studies by participating in the interviews and by completing relevant questionnaires. Patient participants will be adults (phase 1) or young people (phase 2) with mental illness who were admitted to an inpatient ward. Healthcare professionals that will be delivering the intervention and carers/family members of the patients (who consent to be involved) will also be involved in the interview components of the study (patient and carers interviewees can decide whether to be interviewed alone or together).

We will collect data from all consenting patients that are discharged during the baseline and intervention stages, irrespective of whether their inpatient stay began before this period. In the case of an unplanned or early discharge, we will still collect data from consenting patients that have received the intervention, if a patient is not discharged from the pilot ward (due to transfer etc.), they will be ineligible to participant.

Eligibility criteria

Eligible sites
Eligible sites will acute adult inpatient mental health wards. As this is a feasibility study will aim to test on a variety of wards (i.e., female, male or mixed).

Phase 1: SAFER-MH
The eligibility criteria for individual patient participants to take part in the SAFER-MH study reflects the eligibility criteria for services. We will include adult patients aged 18-65 years, informal carers for adult patients with mental health problems aged over 18, healthcare and professionals that are involved in delivering the intervention and informal carers of patients (for process evaluation). All patients will receive the service-level intervention but will choose to participate in the study if they feel they have the capacity consent.

Phase 2: SAFER-YMH
The eligibility criteria to take part in the feasibility study reflects the eligibility criteria for services. Any young person accessing inpatient CAMHS services would be eligible to participate, a parent/carer of a young person accessing inpatient CAMHS services and healthcare Professionals that are involved in delivering the intervention and parents/guardians of the children. All patients will receive the service-level intervention but

For peer review only

will choose to participate in the study if they (their parents/guardians) feel they have the capacity and consent.

Recruitment and Data Collection
Inpatient mental health ward sites

Existing networks will be used to identify eligible services (wards) for the SAFER-MH/YMH studies. The study team will meet with identified services (face-to-face or remotely) to fully explain the purposes of the studies and describe their requirements. Informed consent for services to participate will be provided by the research lead/authorised person in each service, acting as 'guardian' for patients in their care. This will follow agreement with each local ward team clarifying willingness to undertake the SAFER-MH/YMH interventions. Service consent to participate in the study will be formalised through written agreements. The number of services approached, declining, or considered not eligible will be recorded.

Phase 1: SAFER-MH

We aim to recruit three pilot inpatient wards across different trusts in the north west of England to reflect variation of NHS services in England.

Phase 2: SAFER-YMH
We aim to recruit one or two pilot inpatient wards within one trust in the North-West of England. We do not aim to recruit additional trusts because due to funding restrictions, phase 2 must be completed by March 2023.

Patient Participants
All patients on a participating ward will receive the intervention as it is a service level change. However, we will identify patients that are willing to participate in the study assessments (questionnaires and interviews). A ward co-ordinator (e.g., nurse or other health professional) working within the participating services will be asked to co-ordinate handing out questionnaire packs to eligible patients. Potentially eligible patients will be assigned an individual study ID and given an invitation pack close to their expected discharge. This includes an Invitation letter, Patient Information Sheet (PIS), and a Consent to Contact form.

On receipt of an invitation pack, the potential participant will complete the included documents and return to the research practitioner on site. Consent for researchers to contact the potential participant regarding the study will be obtained, in line with the definition outlined in Article 4(11) of the GDPR guidance, "any freely given, specific, informed and unambiguous indication of the data subject's wishes by which he or she, by a statement or by a clear affirmative action, signifies agreement to the processing of personal data relating to him or her" (European Union, 2016). Consent to be contacted with a study recruitment pack will therefore be implied by completion and return of a completed Consent to Contact form.

Assent for young patient participants

Assent will be sought from children / young persons' parents/guardians, or others who are not legally empowered to give consent in phase 2.

Carer/parent/guardian Participants

For peer review only

A study pack will be handed to each patient at discharge, this will include a carer pack, which they can choose to pass on or discard. The pack will include details of the study, paper questionnaires and a paper consent to contact form for interviews, also an online link to assess the documents remotely. For patients who agree to be contacted for an interview, we will ask again if they would like to share the information pack and contact details with their informal carer when contacted.

Professional Participants

Staff participants will be those working directly with patient participants on participating mental health wards (i.e. nurses, health care assistants and doctors). Staff will self-identify to be involved in interviews and questionnaires through posters on the ward and emails sent by the trust research clinician/gatekeeper containing the study information pack (PIS, invitation letter, consent to contact form). All staff that complete the training will be given the opportunity to complete the evaluation survey.

Procedure
Pre-intervention (baseline) phase

This phase will consist of the usual care that the patients in the inpatient wards receive and will last for 6 weeks (see Table 1).. We will assess this current practice by conducting interviews and by inspecting current documentation sheets. These will be completed by one on-site research practitioner. We will perform individual online interviews with the patients, mental health professionals, and carers (relatives) to assess the degree of the satisfaction of the patients and carers as well as their views/thoughts regarding their current discharge plans. At the end of this phase, patients will be asked to complete a series of study questionnaires, including the EQUIP PROM scale (25), which measures the involvement of both the patients and carers in health care planning during/after discharge, The Care Transitions Measure (CTM) (26), and a five-item patient-centred scale focusing on mental health discharge outcomes that were identified as important to patients in previous work (9). Patients, carers, and professionals will also be invited to be interviewed about their experiences of usual care, via the study information pack.

Table 1: Data collection procedures

Baseline Phase (6 weeks)	Training Phase (2 weeks)	Intervention Phase (2 weeks)
Patient/Carer Outcome Measures (n=45):<br>- EQUIP-PROM scale, and,<br>- 5-items regarding general satisfaction about discharge planning	Training evaluation questionnaires	Patient/Carer Outcome Measures (n=45):<br>• EQUIP-PROM scale,<br>• 5-items regarding general satisfaction, and<br>• the Care Transitions Measure (CTM)<br><br>Staff Outcome Measures (n=45):<br>• AIM<br>• IAM<br>• FIM<br>• Adherence to the delivery of the Safer

Interviews with professionals, carers and patients		patient flow bundle will be measured<br><br>Interviews with professionals, carers and patients

Training phase

The training will be a series of self-led short videos and self-help manuals to the pilot ward staff participating in the study. This phase will last for 2 weeks. We aim to use evaluation questionnaires following the training. Interviews in the training phase will explore whether the interventions have been fully comprehended by staff and ensure that we have enough time to solve any problems regarding the training as they arise.

Intervention phase

This phase will last for 6 weeks during which the wards will receive the SAFER-MH and SAFER-YMH patient flow bundle (figure 1). Data regarding the adherence of the mental health professionals to using the SAFER-MH bundle will be collected. Individual online interviews (Zoom) will be conducted with patients and carers (within three months after discharge), and with mental health professionals (within one month after the completion of the 6-week intervention phase) to assess the feasibility and acceptability of the SAFER–MH bundle. Each patient/carer will only be interviewed once, professionals may be interviewed more than once in different stages of the project. At the end of this 6-week phase, patients will be asked to complete the same study scales that were administered during the baseline and which were completed by a different pool of patient participants discharged at the time. We will also administer the three staff outcome measures after the intervention phase is complete.

Figure 1: Timeline of events

Outcomes

The aim of the evaluation will be to determine the feasibility and acceptability of the SAFER-MH and SAFER-YMH interventions by patients, carers and professionals. Additionally, we aim to identify the completion rates and if there are any changes in the key outcomes. As this is a pre-post uncontrolled feasibility study, it will only give us a first indication about the appropriateness of the outcome measures whereas we will primarily focus on whether the SAFER-MH and SAFER-YMH is acceptable and, if so, for whom. We chose 6 week phases, as studies in UK settings indicate average adult length of stays are around 22-36 days and The NHS Mental Health Implementation Plan aims to reduce length of inpatient psychiatric stays to a maximum of 32 days, enabling us to capture a considerable number of patients during the study (27,28).

Quantitative Outcomes

For peer review only - http://bmjopen.bmj.com/site/about/guidelines.xhtml

These will be measured in the baseline phase and intervention phase with different patients. To assess patients and carers perspectives, the quantitative outcomes will be measured using paper/online versions of a series of questionnaires which include: 1) the EQUIP patient reported outcome measure, a 14-item co-designed measure to assess patient/carer views of care planning in mental health (25); 2) a short 4-item version of the Care Transitions Measure (CTM-4) (26), and 3) the five novel items assessing the safety of mental health care transitions (9), properties of these outcome measures can be found in table 2.

To assess professional perspectives, we will use three measures the Acceptability, Appropriateness and Feasibility of Intervention Measure (AIM, IAM and FIM). Each of the three measure contains 4-items to assess professionals' perspectives of a new intervention. These measures will be administered after the intervention phase only (not the baseline). Adherence to the delivery of the SAFER-MH and SAFER-YMH bundle will be measured using a purposefully designed self-report form and recruitment rates will be recorded at each stage.

Table 2: Properties of outcome measures used

Validated Outcome Measure	Psychometric Properties	Scoring system
EQUIP	Scale has acceptable scalability (Ho = .69), reliability (alpha = .92), fit to the Rasch model ($\chi 2(70)$ = 97.25, p = .02), and no differential item functioning or locally dependent items.	It uses a 5-point Likert scale ranging from 1= 'strongly disagree' to 5 = 'strongly agree', with a middle neutral value meaning "neither agree nor disagree." Higher scores indicate higher-perceived quality care planning.
CTM-4	The Cronbach's alpha for CTM-4 is 0.85.	The CTM items are rated on a four-point Likert scale ranging from 1 = 'strongly disagree' to 4 = 'strongly agree', with a fifth response being 'don't know/don't remember/not applicable'. The summarised score (excluding the 'not applicable' scores) is computed as the total sum divided with the number of answered items, minus 1 and multiplied by 100 to get a total score (0–100) for each respondent. Higher scores indicate better patient/carer experience of care transitions
AIM, FIM, IAM	The alphas for 5-item scales were between 0.87 and 0.89. Scale refinement based on measure-specific CFAs and Cronbach alphas using vignette data produced 4-item scales (α's from 0.85 to 0.91). A three-factor CFA exhibited acceptable fit (CFI = 0.96, RMSEA = 0.08) and high factor loadings (0.75 to 0.89), indicating structural validity. ANOVA showed significant main effects, indicating known-groups validity. Test-retest reliability coefficients ranged from 0.73 to 0.88.	1 = Completely disagree, 2 = Disagree, 3 = Neither agree nor disagree, 4 = Agree, 5 =Completely agree Scoring Instructions: Scales can be created for each measure by averaging responses. Scale values range from 1 to 5. Higher scores indicate higher acceptability, appropriateness, and feasibility of the intervention.

For peer review only - http://bmjopen.bmj.com/site/about/guidelines.xhtml

In-depth Qualitative Outcome data

A qualitative process evaluation will be conducted using semi-structured interviews to provide understanding of the study delivery and its potential scale-up. All participants that complete questionnaires will be given the opportunity to volunteer to be interviewed. We aim to interview patients and carers at baseline and intervention, whereas professionals will be interviewed at all three stages (baseline, training, and intervention). All participants (including children) will be remunerated in vouchers for participation.

Data Analysis Plan

We will be following the published extension to Consolidated Standards of Reporting Trials (CONSORT) (29) when reporting the results of this feasibility study. As this is a feasibility study, no formal sample size calculation has been conducted. Our targeted sample size (n=90, phase 1, n=40, phase 2) should be large enough to consider the practicalities of recruitment and delivering the interventions.
Descriptive statistics for intervention feasibility, acceptability and appropriateness will be presented. A chart of participant flow during the whole study will be plotted (CONSORT flow chart) which will include information on the retention/withdrawal (including timing and reasons) of wards during the evaluation. We will also present the number of health practitioners who completed the questionnaires and interviews against the number of professionals working in the wards during the evaluation, and the number of patients/carers completing the questionnaires/interviews against those discharged during the duration of the study. We will also conduct basic psychometric analyses to verify the scale structure and internal consistency of the used questionnaires. For explorative purposes, we will examine differences between the three measures completed by patients recruited during the baseline phase and the measures completed by the patients during the intervention phase. Before starting with the analyses, we will examine if the data is normally distributed. If the data are normally distributed, parametric tests will be used, otherwise non-parametric tests will be used. The significance level for all analyses will be $p \leq 0.05$. The data analyst will be blinded to the phase allocation (baseline or intervention phase).

Qualitative Analysis

We will use a framework approach to qualitative analysis, to inductively code and organise the data and identify emerging themes from the interviews. The frameworks will be based on two underpinning theoretical frameworks commonly used in similar research: the Conceptual Framework for Implementation Fidelity (5 domains), Theoretical Framework of Acceptability (30) and the Theoretical Domains Framework (14 domains). The coding process will follow these steps:

1. Familiarisation (researchers emerging themselves in the data),

2. Identifying a thematic framework (to generate an index of data based on a priori questions and issues raised by participants),

3. Indexing (applying thematic framework to the data using NVivo software),

4. Charting (Rearranging data in line with thematic framework), and

5. Mapping and interpretation (Using the charts to define concepts, map the range and nature of phenomena and find associations between themes).

For peer review only - http://bmjopen.bmj.com/site/about/guidelines.xhtml

Integration of quantitative and qualitative data

The quantitative and qualitative results will be initially considered separately but we aim to examine how they converge or differ when presenting the overall conclusions (31). Furthermore, the qualitative data collected across the two studies will be used to help explain or elaborate on the quantitative data (31).

Patient and public involvement

This study has a patient and public involvement service-user co-researcher who has been involved in the design and delivery of the study (AG). Patient and public involvement has been integral to this study and the development of the intervention which has been based on a series of stakeholder consultations, workshops and consensus methods (9,13,23,32). A core group of five patient and public involvement representatives form a steering committee for this study.

Ethics & Dissemination

Ethical approval was obtained from the National Health Service Cornwall and Plymouth Research Ethics Committee and Surrey Research Ethics Committee (reference: 22/SW/0096 and 22/LO/0404, respectively).

The study findings will be shared with participating inpatient sites in a range of ways to engage different audiences, as well as via presentations at national and international conferences and publishing in open-access peer-reviewed journals. Findings will be presented at a workshop for patients and carers a vlog will be created.

Discussion

This study aims to understand how to improve information sharing and capture between services during patient discharge from mental health hospitals to community services. Extensive stakeholder engagement with patients, carers, mental health professionals and academics has informed the intervention refinement and study co-design. We propose a pragmatic approach to test the feasibility and acceptability of a novel intervention which will add value to the evidence base about how information sharing/capture and patient/carer knowledge and involvement around discharge improves patient safety, health care utilisation and other important patient outcomes.

The findings will have to be interpreted and evaluated considering the limitations of the study design. For example, this is a pre-post uncontrolled study and the potential influence of inpatient settings cannot be controlled for through randomisation. Another limitation is that the interventions have only been developed in English and future work will need to explore how to culturally adapt it. Furthermore, some elements of the intervention may not be suitable for individuals with dementia who often occupy mental health inpatient beds (33), however funding has been obtained to develop this in future work. Finally, the intervention sites will be identified through collaborators, so selection bias must be considered.

The feasibility study will include paper and pdf editable versions of the intervention documents (e.g. social information capture and Patient Written Discharge Plan) which have not been integrated into the Trusts' electronic records yet. Understanding how the intervention documents will fit within the Trusts' electronic records in a key learning task of this feasibility study which will potentially inform a the larger RCT. Similarly, as this is a feasibility study, as

For peer review only - http://bmjopen.bmj.com/site/about/guidelines.xhtml

we are mostly interested in the completion rate of the scales, and the views by patients/carers and staff members about the relevance of these measures to their experiences in relation to hospital discharge. We wont have enough power to generate any meaningful quantitative analysis using our outcome measures and we will not collect quantitative data around readmission rates or adverse events at this stage However, if this study produces convincing evidence that the intervention is feasible/acceptable we will request further funding to conduct a an RCT which will involve more extensive data collection (including routinely collected data on readmissions and adverse events) and a comprehensive quantitative analysis plan a definitive RCT. There is considerable evidence about the importance of community follow-up of patients at post-discharge, Although this is a pre-discharge intervention, there is growing evidence that continuity of care and post-discharge follow-up of patients could enhance safety. Depending on the feedback that we will obtain by the intervention recipients/deliverers during this feasibility study, we will explore whether further adaptations are needed to include post-discharge elements in the intervention protocol.

Improving safety at mental health discharge is a key concern for patients, professionals and policy makers. Therefore, interventions aiming to improve knowledge sharing, shared decision-making and involvement of patients/carers are considered preferable (23,25,34). This study will determine the feasibility and acceptability of a multicomponent intervention to improve involvement of patients and/or carers in discharge planning. This will provide evidence for the acceptability of the SAFER-MH and SAFER-YMH to inform future improvements to scale to a randomised controlled trial, with concurrent process evaluation and economic evaluation.

Authors' contributions: NT and MP had the initial research idea and obtained funding for this study. NT, MP, and IA formulated the research questions and designed the study. AH NT, IA and MP devised the qualitative and statistical analysis plan. NT, MP and IA drafted the protocol. AG led the patient and public involvement components of the study design. JL drafted the protocol and completed ethics documentation/application. RK, CP, SG, NK, CJA, TB, SC, CR substantially contributed to the manuscript by providing review comments and edits and approved the final manuscript.

Funding statement: This work is supported by the NIHR Greater Manchester Patient Safety Translational Research Centre (grant number: PSTRC-2016-003) and NIHR School for Primary Care Research Capacity award 21/22 (grant number C015)Postdoctoral fellowship award. The work. The views expressed are those of the author(s) and not necessarily those of the
NIHR or the Department of Health and Social Care.

Competing interests statement. None

Acknowledgements

We would like to thank all of the patient, public, carer and professional stakeholders who have been involved in the co-design and development of the SAFER-MH intervention to date.

For peer review only - http://bmjopen.bmj.com/site/about/guidelines.xhtml

References

1. Mcmanus S, Meltzer H, Brugha T, Bebbington P, Jenkins R. Adult psychiatric morbidity in England , 2007 Results of a household survey. Appendices and Glossary [Internet]. Health San Francisco. 2007 [cited 2022 Jul 25]. Available from: https://digital.nhs.uk/data-and-information/publications/statistical/adult-psychiatric-morbidity-survey/adult-psychiatric-morbidity-in-england-2007-results-of-a-household-survey
2. NHS England » Mental health [Internet]. [cited 2022 Aug 3]. Available from: https://www.england.nhs.uk/mental-health/
3. Ford T, John A, bmj DG-, 2021 undefined. Mental health of children and young people during pandemic. bmj.com [Internet]. 2021 Mar 10 [cited 2022 Jul 25];372. Available from: https://www.bmj.com/content/372/bmj.n614.full
4. Newlove-Delgado T, McManus S, Sadler K, Thandi S, Vizard T, Cartwright C, et al. Child mental health in England before and during the COVID-19 lockdown. The Lancet Psychiatry. 2021 May 1;8(5):353–4.
5. British Medical Association. The impact of the pandemic on population health and health inequalities BMA Covid Review 5.
6. Forchuk C, Russell G, Kingston-Macclure S, Turner K, Dill S. From psychiatric ward to the streets and shelters. Bombardieri Diaz, Double, Draine, Drake, Drake, Goering, Jones, Koegel, Krupa, Kuno, Lamb, Lamb, Leff, McChesney, Olfson, Philp, Ravignat, Robertson, Rosenheck, Stuart, Sullivan, Susser, Wong C, editor. J Psychiatr Ment Health Nurs [Internet]. 2006;13(3):301–8. Available from: http://ovidsp.ovid.com/ovidweb.cgi?T=JS&PAGE=reference&D=psyc5&NEWS=N&AN=2006-07123-007
7. Walter F, Carr MJ, Mok PLH, Antonsen S, Pedersen CB, Appleby L, et al. Multiple adverse outcomes following first discharge from inpatient psychiatric care: a national cohort study. The Lancet Psychiatry. 2019 Jul 1;6(7):582–9.
8. Healthcare Quality Improvement Partnership. National Confidential Inquiry into Suicide and Safety in Mental Health. ANNUAL REPORT: ENGLAND, NORTHERN IRELAND, SCOTLAND AND WALES 2021 [Internet]. 2021 [cited 2021 Jul 14]. Available from: https://www.research.manchester.ac.uk/portal/en/projects/national-confidential-inquiry-into-suicide-and-safety-in-mental-health(788f9475-cadb-4697-bb82-817638044b7b).html
9. Tyler N, Wright N, Grundy A, Waring J. Developing a core outcome set for interventions to improve discharge from mental health inpatient services: A survey, Delphi and consensus meeting with key stakeholder groups. BMJ Open. 2020.
10. Tyler N, Wright N, Panagioti M, Grundy A, Waring J. What does safety in mental health care transitions mean for service users and other stakeholder groups: An open-ended questionnaire study. Heal Expect. 2020;
11. Tyler N, Wright N, Waring J. Interventions to improve discharge from acute adult mental health inpatient care to the community: systematic review and narrative synthesis. BMC Health Serv Res [Internet]. 2019 Dec 25 [cited 2019 Dec 12];19(1):883. Available from: https://bmchealthservres.biomedcentral.com/articles/10.1186/s12913-019-4658-0
12. Bonsack C, Golay P, Manetti SG, Gebel S, Ferrari P, Besse C, et al. Linking primary and secondary care after psychiatric hospitalization: Comparison between transitional case management setting and routine care for common mental disorders. Bonsack Bonsack, Boyer, Derogatis, Dixon, Goldman, Guelfi, Heggestad, Henderson, Hengartner, Juven-Wetzler, Klinkenberg, Lamb, Nelson, Olfson, Pomini, Puschner, Qin, Steffen, Tomita, Vasiliadis, Vigod, Zusman B, editor. Front Psychiatry [Internet]. 2016;7. Available from: http://ovidsp.ovid.com/ovidweb.cgi?T=JS&PAGE=reference&D=psyc13a&NEWS=N&AN=2016-29356-001

For peer review only - http://bmjopen.bmj.com/site/about/guidelines.xhtml

13. Tyler N, Wright N, Panagioti M, Grundy A, Waring J. What does safety in mental healthcare transitions mean for service users and other stakeholder groups: An open-ended questionnaire study. Heal Expect. 2021 May 1;24(S1):185–94.
14. Keers RN, Williams SD, Vattakatuchery JJ, Brown P, Miller J, Prescott L, et al. Medication safety at the interface: evaluating risks associated with discharge prescriptions from mental health hospitals. J Clin Pharm Ther [Internet]. 2015 Dec [cited 2019 Oct 1];40(6):645–54. Available from: http://www.ncbi.nlm.nih.gov/pubmed/26534824
15. Steffen S, Kösters M, Becker T, Puschner B. Discharge planning in mental health care: A systematic review of the recent literature. Acta Psychiatr Scand [Internet]. 2009;120(1):1–9. Available from: http://ovidsp.ovid.com/ovidweb.cgi?T=JS&PAGE=reference&D=emed12&NEWS=N&AN=354702668
16. Gonçalves-Bradley DC, Lannin NA, Clemson L, Cameron ID, Shepperd S. Discharge planning from hospital. Cochrane Database Syst Rev [Internet]. 2022 Feb 24 [cited 2022 Jul 19];2022(2). Available from: https://www.cochranelibrary.com/cdsr/doi/10.1002/14651858.CD000313.pub6/full
17. Coffey A, Leahy-Warren P, Savage E, Hegarty J, Cornally N, Day MR, et al. Interventions to Promote Early Discharge and Avoid Inappropriate Hospital (Re)Admission: A Systematic Review. Int J Environ Res Public Health [Internet]. 2019 Jul 2 [cited 2022 Mar 29];16(14). Available from: https://pubmed.ncbi.nlm.nih.gov/31295933/
18. Steffen S, Kösters M, Becker T, Puschner B. Discharge planning in mental health care: A systematic review of the recent literature. Acta Psychiatr Scand [Internet]. 2009;120(1):1–9. Available from: https://www.scopus.com/inward/record.uri?eid=2-s2.0-66749126657&doi=10.1111%2Fj.1600-0447.2009.01373.x&partnerID=40&md5=85f563fd242fbfaf95f762a69e7231fe
19. Leppin AL, Gionfriddo MR, Kessler M, Brito JP, Mair FS, Gallacher K, et al. Preventing 30-Day Hospital Readmissions: A Systematic Review and Meta-analysis of Randomized Trials. JAMA Intern Med [Internet]. 2014 Jul 1 [cited 2022 Jul 19];174(7):1095–107. Available from: https://jamanetwork.com/journals/jamainternalmedicine/fullarticle/1868538
20. NHS Improvement. Rapid improvement guide: the SAFER patient flow bundle [Internet]. 2016 [cited 2019 Nov 12]. Available from: https://improvement.nhs.uk/resources/rapid-improvement-guide-safer-patient-flow-bundle/
21. National Institute for Health and Care Excellence. Transition between inpatient mental health settings and community or care home settings | Guidance and guidelines | NICE [Internet]. NICE; 2016 [cited 2018 Dec 20]. Available from: https://www.nice.org.uk/guidance/ng53
22. The SAFER Patient Flow Bundle | Fab NHS Stuff [Internet]. [cited 2022 Aug 22]. Available from: https://fabnhsstuff.net/fab-stuff/28574-the-safer-patient-flow-bundle
23. Tyler N, Planner C, Byrne M, Blakeman T, Keers RN, Wright O, et al. Developing Best Practice Guidance for Discharge Planning Using the RAND/UCLA Appropriateness Method. Front Psychiatry. 2021 Dec 3;12:2204.
24. Tyler, Natasha; Daker-White, Gavin; Grundy, Andrew; Quinliivan, Leah; Armitage, Chris J.; Campbell, Stephen; Panagioti, Maria. The effects of the Covid-19 lockdown on quality and safety in mental health care transitions in England (In Press). BJPsych Open. 2021;
25. Bee P, Gibbons C, Callaghan P, Fraser C, Lovell K. Evaluating and Quantifying User and Carer Involvement in Mental Health Care Planning (EQUIP): Co-Development of a New Patient-Reported Outcome Measure. PLoS One [Internet]. 2016 Mar 1 [cited 2022 Jul 21];11(3):e0149973. Available from: https://journals.plos.org/plosone/article?id=10.1371/journal.pone.0149973
26. Flink M, Tessma M, Småstuen MC, Lindblad M, Coleman EA, Ekstedt M. Measuring

For peer review only - http://bmjopen.bmj.com/site/about/guidelines.xhtml

care transitions in Sweden: validation of the care transitions measure. Int J Qual Heal Care [Internet]. 2018 May 1 [cited 2022 Jul 21];30(4):291. Available from: /pmc/articles/PMC5928451/
27. Crossley N, Sweeney B. Patient and service-level factors affecting length of inpatient stay in an acute mental health service: A retrospective case cohort study. BMC Psychiatry [Internet]. 2020 Sep 7 [cited 2023 Jan 18];20(1):1–9. Available from: https://bmcpsychiatry.biomedcentral.com/articles/10.1186/s12888-020-02846-z
28. National Health Service. NHS Mental Health Implementation Plan 2019/20 – 2023/24. 2019;
29. Saint-Raymond A, Hill S, Martines J, Bahl R, Fontaine O, Bero L. Consort 2010. The Lancet. 2010.
30. Sekhon M, Cartwright M, Francis JJ. Acceptability of healthcare interventions: an overview of reviews and development of a theoretical framework. BMC Heal Serv Res 2017 171 [Internet]. 2017 Jan 26 [cited 2021 Aug 10];17(1):1–13. Available from: https://bmchealthservres.biomedcentral.com/articles/10.1186/s12913-017-2031-8
31. Creswell JW. A Concise Introduction to Mixed Methods Research [Internet]. SAGE Publications; 2014 [cited 2016 Mar 3]. 152 p. Available from: https://books.google.com/books?hl=en&lr=&id=51UXBAAAQBAJ&pgis=1
32. Tyler N, Wright N, Grundy A, Gregoriou K, Campbell S, Waring J. Co-designing a mental health discharge and transitions of care intervention: A Modified Nominal Group Technique. Front Psychiatry. 2020;
33. Edmans BG, Wolverson E, Dunning R, Slann M, Russell G, Crowther G, et al. Inpatient psychiatric care for patients with dementia at four sites in the United Kingdom. Int J Geriatr Psychiatry [Internet]. 2022 Feb 1 [cited 2022 Jul 25];37(2). Available from: https://onlinelibrary.wiley.com/doi/full/10.1002/gps.5658
34. Slade M. Implementing shared decision making in routine mental health care. World Psychiatry. 2017 Jun 1;16(2):146–53.

For peer review only

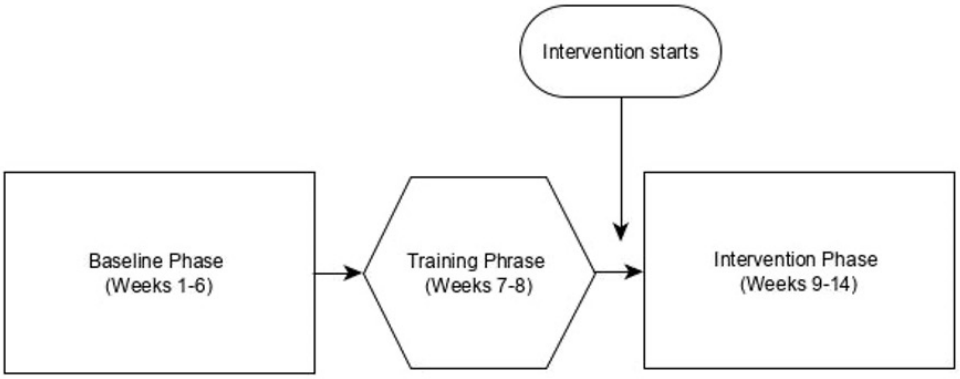

Figure 1: Timeline of events

159x66mm (144 x 144 DPI)
